# Liver Kinase B1 in CD11c^+^ Cells Inhibits Fibrosis in Chronic Pancreatitis via the Oncostatin M Signaling

**DOI:** 10.1002/advs.75018

**Published:** 2026-03-30

**Authors:** Wenqing Zhang, Shan Guo, Yu Zhang, He Ren, Ke Lei, Chenyang Zhao, Qian Yu, Hongqing Luo, Yujing Xiao, Xiaoming Feng, Xiaoyu Li

**Affiliations:** ^1^ Department of Gastroenterology the Affiliated Hospital of Qingdao University Qingdao China; ^2^ Gastrointestinal Cancer Institute (Pancreatic Disease Institute) the Affiliated Hospital of Qingdao University Qingdao China; ^3^ Tumor Immunology and Cytotherapy of Medical Research Center Key Laboratory of Pancreatic Disease Clinical Research (Shandong Province) the Affiliated Hospital of Qingdao University Qingdao China; ^4^ Department of Pathology the Affiliated Hospital of Qingdao University Qingdao China; ^5^ T‐cell Precision Therapy Lab Department of Pathology and Pathophysiology School of Basic Medical Sciences Hangzhou Normal University Hangzhou China

**Keywords:** CCL2/CCR2, chronic pancreatitis, fibrosis, liver kinase B1, oncostatin M

## Abstract

Chronic pancreatitis (CP), characterized by irreversible fibrosis, is a high‐risk factor for pancreatic cancer. Current therapeutic strategies remain inadequate. This study identified a significant increase in CD11c^+^ cells within fibrotic regions of CP patient pancreatic tissues, accompanied by markedly reduced expression of liver kinase B1 (Lkb1) in these cells. Animal experiments confirmed that *Lkb1* deletion in CD11c^+^ cells markedly exacerbated pancreatic fibrosis. Further investigation revealed that *Lkb1* deletion in CD11c^+^ cells drove the differentiation of monocytes into CD11c^+^CD206^+^ mixed‐phenotype macrophages, leading to their accumulation in fibrotic lesions. The CCL2/CCR2 signaling axis was identified as the key pathway mediating the infiltration of these macrophages. Mechanistically, *Lkb1* deletion enhanced STAT3 phosphorylation in CD11c^+^CD206^+^ macrophages, promoted CCL2 secretion, and thereby promoted more CD11c^+^CD206^+^ macrophages to infiltrate. CD11c^+^CD206^+^ macrophages activated pancreatic stellate cells (PSCs) via OSM binding to its receptor OSMR, thereby stimulating CCL2 production of PSCs. This recruits additional monocyte‐derived macrophages to sustain PSC activation and exacerbates extracellular matrix (ECM) deposition. Concurrently, neutralization of OSM suppressed CCL2 secretion from PSCs and markedly attenuated pancreatic fibrosis. This study reveals that *Lkb1* deletion in CD11c^+^ cells exacerbates CP‐associated fibrosis via the OSM and CCL2/CCR2 axis, thereby presenting a potential therapeutic strategy for CP fibrosis.

## Graphical abstract

This study demonstrates significantly reduced Lkb1 expression in CD11c^+^ cells in chronic pancreatitis (CP) patients and animal models. Lkb1 deletion enhances CD11c^+^CD206^+^ macrophage infiltration and reprograms pancreatic stellate cells (PSCs) via OSM signaling. This interaction alters the inflammatory microenvironment and exacerbates PSC activation, highlighting the potential of targeting OSM and CCL2/CCR2 pathways for clinical CP treatment.

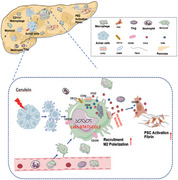

## 1 Introduction

Chronic pancreatitis (CP) is characterized by irreversible, progressive inflammation and fibrosis of pancreatic tissue, representing a high‐risk factor for pancreatic cancer [1, 2, 3]. Current clinical management is primarily symptomatic, focusing on alleviating abdominal pain and diarrhea, with no effective pharmacological therapies targeting pancreatic inflammation and fibrosis [4, 5, 6]. Given the pancreas's distinct immune microenvironment, understanding the underlying pathogenic mechanisms and immune regulatory pathways in CP is essential for developing novel targeted therapies, improving treatment outcomes, and preventing pancreatic cancer onset.

*Liver kinase B1* (*Lkb1*), a serine‐threonine kinase, plays a pivotal role in modulating immune responses and inflammation by regulating the growth, differentiation, and apoptosis of immune cells [7, 8]. *Lkb1* maintains immune homeostasis in dendritic cells (DCs) and influences macrophage function through lipid metabolic reprogramming, endoplasmic reticulum stress, and autophagy regulation [9, 10]. Additionally, *Lkb1* modulates T cell development and activation, impacting inflammatory cytokine production [11, 12, 13]. In approximately 20% of pancreatic ductal adenocarcinoma (PDAC) cases, decreased *Lkb1* expression is significantly associated with poor prognosis [14]. Mice with duct‐specific *Lkb1* deletion exhibit ductal dilation, acinar cell apoptosis, acinar‐ductal metaplasia, and lipomatosis [15]. Our previous studies demonstrated that *Lkb1* negatively regulates the population of regulatory T (Treg) cells across multiple organs. Conditional knockout of *Lkb1* in CD11c^+^ cells leads to excessive proliferation of Treg cells, impaired antigen‐specific T cell responses, and attenuated lipopolysaccharide (LPS)‐induced inflammatory responses [16]. However, the role of *Lkb1* in immune cells during the progression of pancreatic fibrosis development remains unclear.

CD11c, a type I transmembrane protein, has traditionally been recognized as a specific marker for DCs [17, 18, 19]. However, recent studies indicate that bone marrow‐derived CD11c^+^MHCII^+^ cells constitute a heterogeneous population composed of both DCs and monocyte‐derived macrophages. This suggests that CD11c serves not only as a marker for DCs but also as an identifier for monocyte‐derived macrophages [20, 21]. Furthermore, studies across various pathological contexts have revealed that CD11c is widely expressed among macrophage populations. Notably, CD11c^+^ macrophages—rather than dendritic cells (DCs)—exhibit robust pro‐inflammatory and antibacterial functions. These cells highly express inflammatory cytokines, phagocytosis‐related receptors, and MHC‐II molecules, and act as key drivers of inflammation [22, 23, 24, 25, 26]. In CP, M1 macrophages (MHCII^+^TNFα^+^) polarize into M2 macrophages (CD206^+^IL‐10^+^IL‐4Rα^+^) under the influence of interleukin (IL)‐4 and IL‐13, leading to a predominance of M2 macrophages, which promote fibrosis [27, 28, 29]. However, the presence and role of CD11c^+^ macrophages in CP pancreatic tissue remain poorly understood, and their potential involvement in CP‐related inflammation and fibrosis warrants further investigation.

This study identified a population of CD11c^+^ cells within the fibrotic regions of pancreatic tissues from patients with CP. These cells were characterized by significantly downregulated expression of Lkb1 compared to those in normal tissues, suggesting a potential regulatory role for Lkb1 in CD11c^+^ cells in the pathogenesis of CP. Using a CP mouse model with CD11c^+^ cell‐specific knockout of *Lkb1*, we demonstrated that Lkb1 acts as a novel inflammatory suppressor that attenuates pancreatic fibrosis progression. Mechanistically, *Lkb1* deletion enhanced STAT3 phosphorylation in CD11c^+^CD206^+^ macrophages, thereby promoting CCL2 secretion. Furthermore, CD11c^+^CD206^+^ macrophages activate PSCs and induce CCL2 production through the binding of OSM (oncostatin M) to OSMR on PSCs. Inhibition of the CCL2/CCR2 axis and administration of an OSM‐neutralizing antibody validated their critical involvement in mediating Lkb1‐dependent suppression of CD11c^+^CD206^+^ macrophage infiltration and fibrotic progression in CP. These findings highlight the therapeutic potential of targeting the OSM signaling and CCL2/CCR2, providing new insights for treatment strategies in CP.

## 2 Results

### 2.1 Lkb1 Deletion in CD11c^+^ Cells Drives Fibrosis and Inflammation in Chronic Pancreatitis

**FIGURE 1:**
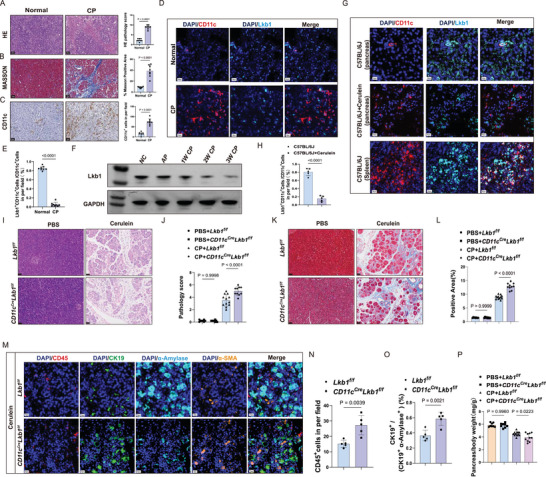
*Lkb1* deletion in CD11c^+^ macrophages drives fibrosis and inflammation in CP. (A) Representative images of pancreatic H&E staining. Scale bar, 100 µm. *n* = 8. (B) Representative images of pancreatic Masson's staining. Scale bar, 100 µm. *n* = 8. (C) Representative immunohistochemistry (IHC) staining images of CD11c in pancreatic tissue. Scale bar, 50 µm. *n* = 8. (D,E) Representative immunofluorescence images of CD11c, Lkb1, and DAPI co‐staining in CP and normal pancreatic tissues. Scale bar, 50 µm. *n* = 8. (F) Western blot analysis of Lkb1 expression in pancreatic CD11c^+^ cells at multiple time points after disease induction. *n* = 3. (G,H) Representative immunofluorescence images of CD11c, Lkb1, and DAPI co‐staining in pancreatic tissues from control and CP model WT mice, as well as in splenic tissues from control WT mice. Scale bar, 50 µm. *n* = 5. (I–L) Representative images of pancreatic H&E staining and Masson's staining. Scale bar, 100 µm. From left to right: *n* = 8; *n* = 8; *n* = 12; *n* = 10. (M) Representative immunofluorescence images of co‐staining for CD45, CK19, α‐Amylase, α‐SMA, and DAPI in the pancreas with (N,O) statistical analysis. Scale bar, 50 µm. *n* = 5. (P) Relative pancreatic weight (pancreas/body weight). From left to right: *n* = 8; *n* = 8; *n* = 12; *n* = 10. Data are means ± SEM. Unpaired Student's *t*‐tests (A, B, C, E, H, N, O) were used to evaluate statistical significance. Data were analyzed using one‐way ANOVA (J, L, P) with the Tukey test.

Comparative analysis of pancreatic tissue specimens from CP patients and organ donors revealed a pronounced increase in fibrosis within the CP cohort (Figure 1A,B). Quantitative immunohistochemical (IHC) assessment demonstrated a significant enrichment of CD11c^+^ immune cells predominantly localized to fibrotic regions in CP tissues (Figure 1C). Further immunofluorescence analysis demonstrated a significant downregulation in Lkb1 expression exclusively within the CD11c^+^ cell population in CP tissues (Figure 1D,E), indicating an inverse correlation between Lkb1 expression levels and the extent of pancreatic fibrosis. We established a CP animal model and dynamically tracked Lkb1 expression in pancreatic CD11c^+^ cells at multiple time points after disease induction, including 1 day, 1 week, 2 weeks, and 3 weeks (Figure 1F). We found that Lkb1 expression in CD11c^+^ cells progressively decreased with increasing duration of CP induction. Collectively, these results imply that Lkb1 may serve as a negative regulator in the fibrogenic processes mediated by the CD11c^+^ cell subset.

To elucidate the functional role of *Lkb1* in CP, we employed genetically engineered murine models. Specifically, *CD11c^Cre^Lkb1^f/f^* mice and their *Lkb1^f/f^* littermate controls were utilized. First, we established a CP model in Wild‐type (WT) mice. Immunofluorescence analysis revealed that in the context of CP, Lkb1 expression was downregulated in CD11c^+^ cells within the pancreatic tissue of WT mice (Figure 1G,H). Subsequently, we induced CP in both *CD11c^Cre^Lkb1^f/f^* and *Lkb1^f/f^* mice; histopathological analysis revealed that *CD11c^Cre^Lkb1^f/f^* mice demonstrated significantly exacerbated CP pathology, as evidenced by severe pancreatic acinar atrophy, robust immune cell infiltration, and extensive fibrotic remodeling (Figure 1I–L). These histopathological alterations were accompanied by enhanced PSC activation and increased collagen deposition (Figure 1M–O; Figure S1A,B). Quantitative analysis further revealed a marked reduction in the pancreatic weight‐to‐body weight ratio in *CD11c^Cre^Lkb1^f/f^* mice, concomitant with elevated expression of profibrotic mediators including *TGF‐β1*, *PDGF‐β*, and the myofibroblast marker *α‐SMA*, within affected CP tissues (Figure 1P, Figure S1C,D). Comprehensive transcriptomic profiling of CP‐affected pancreatic tissues identified that *Lkb1* deletion specifically in CD11c^+^ cells specifically potentiated inflammation‐associated signaling pathways (Figure S1E,F and Table S1). Notably, conditional *Lkb1* deletion in CD11c^+^ cells did not compromise normal pancreatic architecture, animal survival, or reproductive function across various age groups, with no evidence of spontaneous pancreatitis (Figure S2A–E). Comparative analysis in an acute pancreatitis (AP) model using both *Lkb1^f/f^* and *CD11c^Cre^Lkb1^f/f^* mice demonstrated no differences in AP severity (Figure S2F,G). Collectively, these findings demonstrate that *Lkb1* deletion in CD11c^+^ cells plays a critical role in amplifying inflammatory responses and promoting fibrogenic processes during CP pathogenesis.

### 2.2 scRNA‐Seq Reveals Alterations in the Pancreatic Immune Microenvironment in CP with *Lkb1* Deletion in CD11c^+^ Cells

**FIGURE 2:**
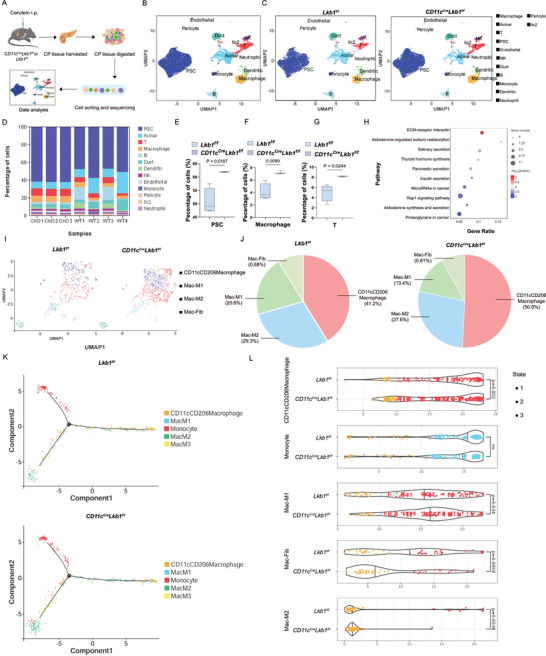
*Lkb1* deletion in CD11c^+^ cells modulates the inflammatory microenvironment of CP. (A) Overview of the CP mouse model and scRNA‐seq workflow. (B) UMAP plots illustrating distinct cell populations in the pancreas of CP mice. (C) UMAP plot displaying distinct cell populations in CP pancreatic tissues of *Lkb1^f/f^* and *CD11c^Cre^Lkb1^f/f^* mice. (D) Percentage of cells of different cell types in CP pancreatic tissues of *Lkb1^f/f^* and *CD11c^Cre^Lkb1^f/f^* mice. (E–G) Differences in the frequency of PSCs、T cells or macrophages as a percentage of total cells. (H) KEGG enrichment analysis of upregulated genes in PSCs. (I) UMAP plot of macrophage subclusters in CP pancreatic tissue. (J) Percentage of cells of different macrophage subclusters in macrophages of *Lkb1^f/f^* and *CD11c^Cre^Lkb1^f/f^* mice. (K) Trajectory plots display pseudo‐time trajectory analysis of various macrophage subpopulations derived from monocytes. (L) Violin plot showing the progression of cells from *Lkb1^f/f^* and *CD11c^Cre^Lkb1^f/f^* mice along the determined trajectory in pseudotime. (A–L) *Lkb1^f/f^* (*n* = 4) and *CD11c^Cre^Lkb1^f/f^* (*n* = 3) mice (each dot represents an animal). Data are means ± SEM. Unpaired Student's *t*‐tests (E–G) were used to evaluate statistical significance.

To further elucidate the functional role of *Lkb1* in CD11c^+^ cells during CP, we performed single‐cell RNA sequencing (scRNA‐seq) on pancreatic tissue of *Lkb1^f/f^* and *CD11c^Cre^Lkb1^f/f^* mice (Figure 2A). Uniform manifold approximation and projection (UMAP) analysis was employed to identify and visualize distinct cellular clusters in two dimensions. Twelve unique cell clusters were delineated based on lineage‐specific transcription profiles (Figure 2B–D, Figure S3A). Notably, a significant increase in the proportion of PSCs, macrophages, and T cells was observed in *CD11c^Cre^Lkb1^f/f^* mice compared to controls (Figure 2E–G).

Further characterization of these cell populations, coupled with Kyoto Encyclopedia of Genes and Genomes (KEGG) pathway enrichment analysis of differentially upregulated genes in PSCs, revealed a significant enrichment of biological processes within the ECM‐receptor interaction pathway (Figure 2H, Table S2). These findings suggest that *Lkb1* deletion in CD11c^+^ cells promotes ECM production in PSCs during CP. Further analysis of the scRNA‐seq data revealed distinct transcriptional profiles of T cell subsets in *Lkb1^f/f^* and *CD11c^Cre^Lkb1^f/f^* mice. Notably, *CD11c^Cre^Lkb1^f/f^* mice exhibited a higher percentage of Foxp3^+^CD4^+^ regulatory T cells (Treg) (Figure S3B–D). Comprehensive macrophage clustering identified four predominant subtypes: classically activated (M1) macrophages (Mac‐M1), characterized by the expression of proinflammatory markers including *Cd80* and *Il1β*; alternatively activated (M2) macrophages (Mac‐M2), enriched for anti‐inflammatory and tissue‐remodeling genes such as *Mrc1* and *Cd36*; fibroblast‐like macrophages (Mac‐Fib), exhibiting a fibrogenic signature with high expression of extracellular matrix components *Col1a1* and *Col3a1*; and a unique CD11c^+^CD206^+^ macrophages (Figure 2I, Figure S4A–D). Notably, *CD11c^Cre^Lkb1^f/f^* mice exhibited a significantly higher percentage of CD11c^+^CD206^+^ macrophages (Figure 2J). To gain further insight into the potential functional heterogeneity among these four macrophage subsets, we performed KEGG pathway enrichment analysis on their upregulated differentially expressed genes. Inflammatory responses were observed in both Mac‐M1 and Mac‐M2 subsets. Specifically, Mac‐M2 showed enrichment in pathways such as Phagosome and Complement and coagulation cascades, whereas Mac‐M1 was primarily associated with Glycerolipid metabolism. Mac‐Fib appeared to be enriched in ECM‐receptor interaction and Protein processing in the endoplasmic reticulum. The CD11c^+^CD206^+^ macrophages were enriched in pathways including Fc gamma R‐mediated phagocytosis and the Chemokine signaling pathway (Figure S4E). Differential gene expression profiling further revealed significant upregulation of mature macrophage signature genes, such as *Runx3* and *Cx3cr1* in the *CD11c^Cre^Lkb1^f/f^* mouse model (Figure S4F).

Given the observed increase in macrophages in *CD11c^Cre^Lkb1^f/f^* mice, we performed pseudotime trajectory analysis using Monocle 2 to reconstruct the developmental pathway and model the differentiation process, thereby exploring the role of Lkb1 in monocyte differentiation (Figure 2K). Pseudotime analysis revealed that, compared with monocytes derived from *Lkb1^f/f^* mice, those from *CD11c^Cre^Lkb1^f/f^* mice exhibited a significant bias in their differentiation potential towards various macrophage lineages. Notably, the most pronounced difference was observed in the commitment to the CD11c^+^CD206^+^ macrophage subpopulation (Figure 2L). Collectively, these findings indicate that *Lkb1* deletion in CD11c^+^ cells primarily promotes the lineage commitment of monocytes towards the CD11c^+^CD206^+^ macrophage phenotype. In conclusion, *Lkb1* deletion in CD11c^+^ cells significantly remodels the pancreatic microenvironment in CP, an effect predominantly mediated through the enhanced infiltration of CD11c^+^CD206^+^ macrophages.

### 2.3 *Lkb1* Deletion in CD11c^+^ Cells Promotes a Predominant Infiltration of CD11c^+^CD206^+^ Macrophages in CP

**FIGURE 3:**
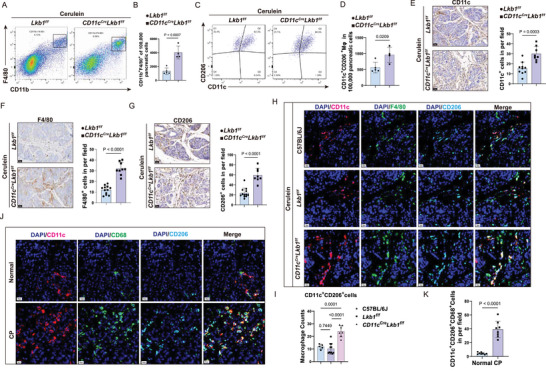
*Lkb1* deletion in CD11c^+^ cells promotes an increase in CD11c^+^CD206^+^ macrophages in CP. (A,B) Representative flow cytometry plots and the absolute number of macrophages in the pancreas. From left to right: *n* = 5; *n* = 4. (C,D) Representative flow cytometry plots and the absolute number of CD11c^+^CD206^+^ macrophages in the pancreas. From left to right: *n* = 5; *n* = 4. (E–G) Representative IHC staining images and statistical analysis of CD11c (*n* = 10; *n* = 8), F4/80 (*n* = 12; *n* = 9), and CD206 (*n* = 12; *n* = 8). Scale bar, 50 µm. (H,I) Representative immunofluorescence images of co‐staining for F4/80, CD206, CD11c, α‐SMA, and DAPI in the pancreas. Scale bar, 50 µm. *n* = 8. (J,K) Representative immunofluorescence images of CD68, CD206, CD11c, and DAPI co‐staining in pancreatic tissues of patients with clinical CP. Scale bar, 50 µm. *n* = 8. Data are means ± SEM. Unpaired Student's *t*‐tests were used to evaluate statistical significance.

The systemic impact of *Lkb1* deletion in CD11c^+^ cells on the CP pancreatic immune microenvironment was comprehensively investigated. Flow cytometry analysis demonstrated a significant increase in the absolute count of macrophages within the pancreatic tissues of *CD11c^Cre^Lkb1^f/f^* mice relative to *Lkb1^f/f^* mice (Figure 3A,B, Figure S5A). Additionally, the proportion of CD11c^+^CD206^+^ macrophages exhibited a significant increase, constituting the predominant macrophage subpopulation (Figure 3C,D). IHC staining validated the presence of CD11c^+^, F4/80^+^, and CD206^+^ cellular infiltrates within CP tissues, with particular enrichment in the pancreatic interstitial compartments (Figure 3E–G). Multiplex immunofluorescence analysis further identified co‐localized CD11c^+^F4/80^+^CD206^+^ cells within fibrotic regions of the pancreatic interstitium, displaying markedly enhanced infiltration in *CD11c^Cre^Lkb1^f/f^* mice (Figure 3H,I). scRNA‐seq revealed the expression patterns of various macrophage‐associated marker genes (Figure S5B). Flow cytometry analysis revealed that *Lkb1* deletion in CD11c^+^ cells did not result in significant alterations in CD11c^+^ DCs (Figure S5C). Concurrently, these fibrotic areas exhibited minimal detection of classically activated (M1‐like) iNOS^+^ macrophages (Figure S6A). Clinically, CP patients exhibiting reduced Lkb1 expression in CD11c^+^ cells demonstrated a significant accumulation of CD11c^+^CD206^+^CD68^+^ macrophages (Figure 3J,K). These findings indicate that *Lkb1* deletion in CD11c^+^ cells primarily promotes predominant infiltration of CD11c^+^CD206^+^ macrophages.

Previous studies have demonstrated that *Lkb1* deletion in CD11c^+^ cells induces spontaneous Treg expansion in multiple organs, leading to a systemic immunosuppressive phenotype [16]. Interestingly, however, no such spontaneous Treg expansion was detected in the pancreatic tissue of *CD11c^Cre^Lkb1^f/f^* mice (Figure S6B), implying that *Lkb1* may exert tissue‐specific immunomodulatory effects in the pancreas, likely influenced by its distinctive immune microenvironment. Under CP conditions, *CD11c^Cre^Lkb1^f/f^* mice displayed markedly enhanced infiltration of CD4^+^ T cell, particularly Treg cells, whereas CD8^+^ T cell infiltration remained unaltered (Figure S6C–H). Consistent with these findings, human CP samples also exhibited a similar increase in Treg infiltration (Figure S6I,J). In line with these observations, no significant changes were noted in the infiltration of natural killer (NK) or B cells (Figure S6K–N). Taken together, these results indicate that within the CP microenvironment, *Lkb1* deletion in CD11c^+^ cells primarily promotes the recruitment of immunomodulatory CD11c^+^CD206^+^ macrophages into the pancreas, while simultaneously enhancing Treg accumulation to maintain local immune homeostasis.

### 2.4 *Lkb1* Deletion in CD11c^+^ Cells Promotes Predominant Infiltration of CD11c^+^CD206^+^ Macrophages via the CCL2/CCR2 Axis

**FIGURE 4:**
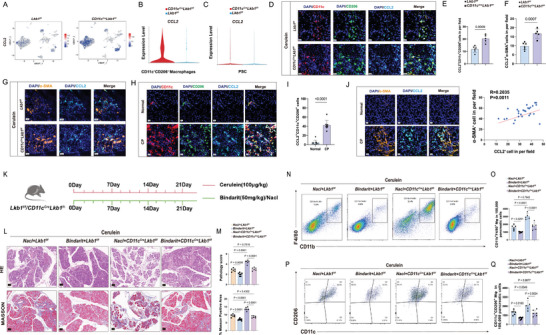
*Lkb1* deletion in CD11c^+^ cells promotes CCL2 secretion and recruitment of macrophages. (A) UMAP plots showing the gene expression and distribution of CCL2 in the subpopulation. *Lkb1^f/f^* (*n* = 4) and *CD11c^Cre^Lkb1^f/f^* (*n* = 3) mice. (B,C) Violin plots showing the expression levels of CCL2 in CD11c^+^CD206^+^ macrophages and PSCs. *Lkb1^f/f^* (*n* = 4) and *CD11c^Cre^Lkb1^f/f^* (*n* = 3) mice. (D,E) Immunofluorescence images showing co‐staining of CD11c, CD206, CCL2, α‐SMA, and DAPI in mouse pancreatic tissue. Scale bar, 50 µm. From left to right: *n* = 6; *n* = 5. (F,G) Immunofluorescence images showing co‐staining of α‐SMA, CCL2, and DAPI in mouse pancreatic tissue. Scale bar, 50 µm. From left to right: *n* = 6; *n* = 5. (H,I) Immunofluorescence images showing co‐staining of CD11c, CD206, CCL2, α‐SMA, and DAPI in human pancreatic tissue. Scale bar, 50 µm. *n* = 8. (J) Statistical chart of correlation between CCL2^+^ cells and α‐SMA^+^ cells. (K) Schematic representation of the experimental model using CCL2 inhibitors. (L) Representative images of pancreatic tissue stained with H&E staining and Masson's staining, and (M) pathological scores indicating statistical analysis. Scale bar, 100 µm. From left to right: *n* = 6; *n* = 7; *n* = 6; *n* = 6. (N) Representative flow cytometry plots and (O) absolute numbers of total macrophages. From left to right: *n* = 6; *n* = 6; *n* = 5; *n* = 6. (P) Representative flow cytometry plots and (Q) absolute numbers of CD11c^+^CD206^+^ macrophages. From left to right: *n* = 6; *n* = 6; *n* = 5; *n* = 6. Data are means ± SEM. Unpaired Student's *t*‐tests (E,F,I) were used to evaluate statistical significance. Data were analyzed using one‐way ANOVA (M,O,Q) with the Tukey test.

Given the elevated abundance of macrophages, we investigated the chemokine signaling pathways implicated in monocyte and macrophage recruitment. scRNA‐seq analysis demonstrated that *Lkb1* deletion induced a marked upregulation of the pivotal monocyte/macrophage chemokine CCL2 (Figure 4A). Further dissection revealed that the enhanced CCL2 secretion in pancreatic tissue predominantly originated from CD11c^+^CD206^+^ macrophages and PSCs (Figure 4B,C), with negligible contribution from CD11c^+^ DCs (Tables S3,S4). RT‐qPCR validated a significant increase in *CCL2* mRNA expression (Figure S7A), which was further corroborated by IHC staining, revealing elevated CCL2 protein levels in *CD11c^Cre^Lkb1^f/f^* mice (Figure S7B). In contrast, no substantial alterations were detected in other chemokines, including *CCL3* and *CCL5* (Figure S7C). Immunofluorescence analysis confirmed the co‐localization of CCL2 with CD11c^+^CD206^+^ macrophages and PSCs (Figure 4D–G). Moreover, in CP patient samples, fibrotic regions exhibited heightened CCL2 secretion by CD11c^+^CD206^+^ cells and PSCs (Figure 4H,I), with a robust positive correlation between CCL2 expression levels and the degree of pancreatic fibrosis (Figure 4J). Collectively, these findings indicate that *Lkb1* deletion in CD11c^+^ cells potentiates CCL2 production by CD11c^+^CD206^+^ macrophages and PSCs.

To evaluate the involvement of the CCL2/CCR2 axis in *Lkb1* deletion‐induced pancreatic fibrosis, we administered Bindarit (a selective inhibitor of CCL2 synthesis) and RS504393 (a potent CCR2 antagonist) to assess their effects on immune cell infiltration and fibrosis progression in the context of *Lkb1* deletion in CD11c^+^ cells (Figure 4K, Figure S8A). While *CD11c^Cre^Lkb1^f/f^* mice exhibited exacerbated inflammatory fibrosis compared to *Lkb1^f/f^* controls following cerulein challenge, therapeutic intervention with either Bindarit or RS504393 not only prevented the *Lkb1* deletion‐mediated worsening of fibrosis but also reversed the disparity in inflammatory fibrotic pathology between the two genotypes (Figure 4L,M, Figure S8B–D). To further elucidate the impact of these pharmacological agents on the pancreatic immune microenvironment, flow cytometry analysis demonstrated that CCL2 inhibition effectively reversed the *Lkb1* deletion‐associated increase in CD11c^+^CD206^+^ macrophage subsets (Figure 4N–Q). Multiplex immunofluorescence assays revealed that CCR2 blockade significantly attenuated the *Lkb1* deletion‐induced expansion of M2 macrophages in CD11c^+^ cells (Figure S8E,F). Additionally, immunofluorescence analysis confirmed that CCL2 inhibition markedly diminished the accumulation of Treg cells associated with *Lkb1* deletion (Figure S8A,B). In summary, these findings indicate that therapeutic inhibition of CCL2 production or blockade of CCR2 effectively suppresses the *Lkb1*‐deficient‐mediated increase in CD11c^+^CD206^+^ macrophage infiltration within CD11c^+^ cells during CP, thereby attenuating CP‐associated fibrosis.

### 2.5 *Lkb1* Deletion in CD11c^+^ Cells Promotes CCL2 Secretion by CD11c^+^CD206^+^ Macrophages Through JAK2‐STAT3

**FIGURE 5:**
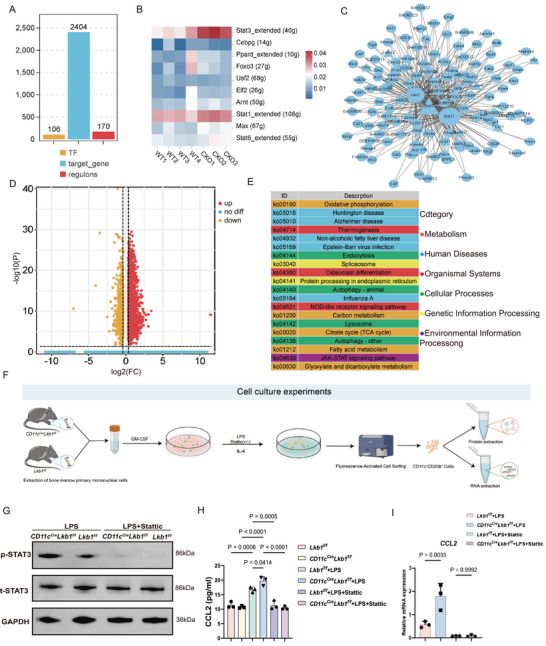
*Lkb1* regulates the expression of its target gene CCL2 in CD11c^+^CD206^+^ macrophages by inhibiting STAT3 phosphorylation. (A) Single‐cell regulatory network inference and clustering analysis showing the number of regulons, the number of TFs, and the number of target genes obtained by prediction. (B) Regulon activity values of subpopulations using a heatmap to demonstrate the heterogeneity and distribution characteristics of regulons in subpopulations. (C) WGCNA‐based gene co‐expression network analysis. (D) Volcano plot showing differentially expressed genes in CD11c^+^CD206^+^ macrophages. (E) KEGG enrichment analysis of upregulated differential genes in CD11c^+^CD206^+^ macrophages. (F) Schematic diagram of cell culture. (G) Western blot analysis of p‐STAT3 and t‐STAT3 in CD11c^+^CD206^+^ cells. (H) CCL2 secretion level in cell culture supernatants. (I) mRNA expression level of *CCL2*. (A–E) *Lkb1^f/f^* (*n* = 4) and *CD11c^Cre^Lkb1^f/f^* (*n* = 3) mice, (F–I) *n* = 3. Data are means ± SEM. Data were analyzed using one‐way ANOVA (H,I) with the Tukey test.

To further elucidate the molecular mechanisms underlying the regulation of CCL2 by *Lkb1* deletion in CD11c^+^ cells within a CP mouse model, we performed comprehensive transcriptomic and proteomic analyses on CD11c^+^ cells isolated from pancreatic tissues of CP mice. Integrative analysis of these omics data with scRNA‐seq data enabled cross‐prediction of transcription factors (TFs), revealing STAT3 as a pivotal downstream effector of *Lkb1* (Figure 5A,B, Figure S10A–F and Table S5). This finding was further substantiated through Weighted Gene Co‐expression Network Analysis (WGCNA)‐based gene co‐expression network analysis (Figure 5C). Nevertheless, the precise mechanism by which *Lkb1* modulates STAT3 activation remains to be elucidated. Notably, KEGG pathway enrichment analysis of scRNA‐seq data from CD11c^+^CD206^+^ macrophages implicated the JAK‐STAT signaling pathway as a potential mediator of *Lkb1* function (Figure 5D,E).

To mechanistically investigate this relationship, we stimulated bone marrow‐derived monocytes from *Lkb1^f/f^* and *CD11c^Cre^Lkb1^f/f^* mice with GM‐CSF to differentiate them into CD11c^+^ cells, and CD11c^+^CD206^+^ cell populations were subsequently isolated (Figure 5F, Figure S11A). Further investigations revealed that *Lkb1* deletion also significantly enhanced the expression levels of fibrosis‐related factors, including TGF‐β1, in CD11c^+^CD206^+^ macrophages (Figure S11B–G). These findings indicate that *Lkb1* deletion not only drives phenotypic polarization towards a CD11c^+^CD206^+^ state but also functionally augments the pro‐fibrotic capacity of this macrophage subset. Western blot analysis demonstrated that *Lkb1* deletion resulted in significant upregulation of both phosphorylated STAT3 (p‐STAT3) and its upstream regulator phosphorylated JAK2 (p‐JAK2) (Figure 5G, Figure S12A,B), indicating that *Lkb1* deletion enhances STAT3 phosphorylation in CD11c^+^CD206^+^ macrophages. Given that CCL2 is a well‐established downstream target of STAT3 [30], we confirmed through ELISA and RT‐qPCR assays that the *Lkb1* deletion‐induced elevation in both CCL2 expression and secretion could be effectively reversed by treatment with the STAT3 phosphorylation inhibitor Static (Figure 5H,I). These findings collectively establish that *Lkb1* regulates CCL2 expression and secretion in a STAT3 phosphorylation‐dependent manner.

### 2.6 The OSM Signaling Pathway Mediates the Interaction Between CD11c^+^CD206^+^ Macrophages and PSCs

**FIGURE 6:**
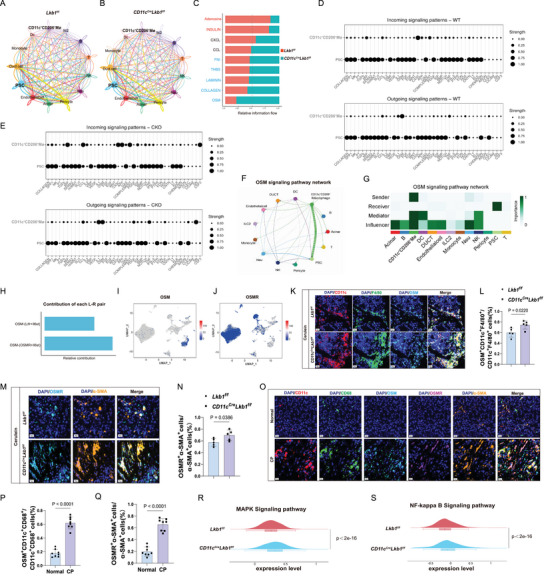
*Lkb1* in CD11c^+^ cells mediates the interaction between CD11c^+^CD206^+^ macrophages and PSCs through the OSM signaling pathway. (A,B) Multi‐subpopulation network diagrams showing the number of ligand‐receptor pairs and expression abundance or probability information in cellular subpopulation pairs. (C) Bar graphs showing the proportion of CD11c^+^CD206^+^ macrophage‐PSCs interacting with signaling pathways in the two groups. (D,E) Dot plots of the cell‐cell communication between PSCs and CD11c^+^CD206^+^ macrophages. (F) Network map of cellular communication at the level of the OSM signaling pathway in the *CD11c^Cre^Lkb1^f/f^* group. (G) Heatmap showing the relative importance of each cell group calculated based on four network‐centered metrics of OSM signaling in CP mice of the *CD11c^Cre^Lkb1^f/f^* group. (H) Bar plots showing the contribution of each ligand‐receptor pair to the OSM signaling pathway. Characterization maps of (I) OSM and (J) OSMR gene expression. (K) Representative immunofluorescence images of co‐staining for CD11c, F4/80, OSM, and DAPI in the pancreas with (L) statistical analysis. Scale bar, 50 µm. (M) Representative immunofluorescence images of co‐staining for OSMR, α‐SMA, and DAPI in the pancreas with (N) statistical analysis. Scale bar, 50 µm. (O) Representative immunofluorescence images of co‐staining for CD11c, CD68, OSM, OSMR, α‐SMA, and DAPI in human CP and normal pancreatic tissues with (P,Q) statistical analysis. Scale bar, 50 µm. Mountain range plots showing gene expression in PSCs associated with the (R) MAPK or (S) NF‐κB signaling pathways. (A–J,R,S) *Lkb1^f/f^* (*n* = 4) and *CD11c^Cre^Lkb1^f/f^* (*n* = 3) mice, K‐N) *n* = 5, (O–Q) *n* = 8. Data are means ± SEM. Unpaired Student's *t*‐tests (L–Q) were used to evaluate statistical significance.

To further elucidate the molecular mechanisms underlying the enhanced secretion of CCL2 by PSCs, we performed a systematic analysis of cellular communication between CD11c^+^CD206^+^ macrophages and PSCs. CellChat analysis revealed that *Lkb1* deletion significantly enhanced CD11c^+^CD206^+^ macrophages intercellular interactions with PSCs, primarily mediated by secreted signaling factors (Figure 6A,B). Specifically, *Lkb1* deletion led to a marked upregulation of the OSM (oncostatin M) signaling pathway in CD11c^+^CD206^+^ macrophages along with multiple fibrosis‐associated pathways, including those involving COLLAGEN, LAMININ, FN1, and THBS, while the CXCL and CCL signaling pathways remained unaffected (Figure 6C, Figure S13A–C). As a key member of the IL‐6 cytokine family, OSM exerts its biological effects primarily through binding to the Oncostatin‐M receptor (OSMR) [31, 32]. Our data demonstrated that the secreted factor‐mediated signaling network between CD11c^+^CD206^+^ macrophages and PSCs exhibited dynamic bidirectional communication patterns, with both outgoing and incoming signaling activities (Figure 6D,E). Further dissection of the OSM‐OSMR signaling axis identified CD11c^+^CD206^+^ macrophages as the predominant source of OSM, whereas PSCs served as the primary target cells (Figure 6F,G). This interaction was mediated by two key ligand‐receptor pairs, involving OSMR and the IL‐6 signal transducer (IL6ST) (Figure 6H, Figure S13D). Immunofluorescence imaging and UMAP‐based cell clustering analyses corroborated these findings, showing that OSM is predominantly expressed in CD11c^+^CD206^+^ macrophages, while its cognate receptor OSMR is enriched in PSCs (Figure 6I–Q). Notably, OSM signaling can trigger downstream activation of the mitogen‐activated protein kinase (MAPK) cascade [33]. Consistent with this, our results demonstrated that *Lkb1* deletion leads to a significant upregulation of key components of the MAPK and NF‐κB signaling pathways in PSCs (Figure 6R,S). These results demonstrate that in the context of CP, *Lkb1* deletion in CD11c^+^CD206^+^ cells further promotes their interaction with PSCs via the OSM‐OSMR signaling axis.

### 2.7 *Lkb1* Deletion in CD11c^+^ Cells Leads to CD11c^+^CD206^+^ Macrophages Increased Activation of PSC and CCL2 Secretion Through the OSM‐OSMR Signaling Axis

**FIGURE 7:**
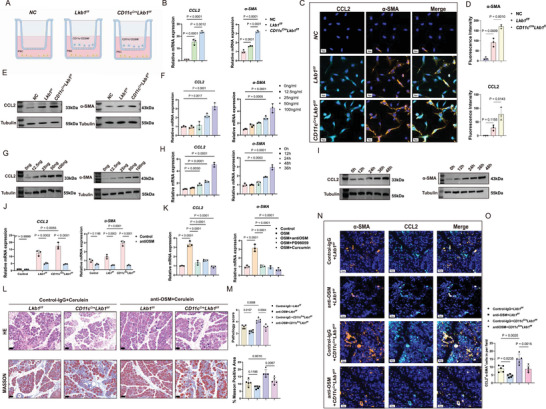
*Lkb1* deletion promotes PSC activation and CCL2 secretion via OSM signaling. (A) Schematic diagram of the co‐culture system. B) mRNA expression levels of *CCL2* and *α‐SMA* in PSCs following co‐culture. (C,D) Representative immunofluorescence images showing co‐staining for CCL2, α‐SMA, and DAPI in PSCs after co‐culture. Scale bar, 50 µm. (E) Western blot analysis of CCL2 and α‐SMA in PSCs following co‐culture. (F) mRNA expression levels of *CCL2* and *α‐SMA* in PSCs following 36‐hour OSM treatment at varying concentrations. (G) Western blot analysis of CCL2 and α‐SMA in PSCs following 36‐hour OSM treatment at varying concentrations. (H) mRNA expression levels of *CCL2* and *α‐SMA* in PSCs after stimulation with OSM (50 ng/mL) for different durations. (I) Western blot analysis of CCL2 and α‐SMA in PSCs after stimulation with OSM (50 ng/mL) for different durations. (J) Neutralization of OSM (1 µg/mL) in the co‐culture system and assessment of the mRNA expression levels of *CCL2* and *α‐SMA* in PSCs. (K) mRNA expression levels of *CCL2* and *α‐SMA* in PSCs pretreated with MAPK (20 µmol/mL) or NF‐κB (20 µmol/mL) inhibitors during OSM exposure. (L,M) Representative images of pancreatic H&E staining and Masson's staining. Scale bar, 100 µm. (N,O) Immunofluorescence images showing co‐staining for CCL2, α‐SMA, and DAPI in mouse pancreatic tissue. Scale bar, 50 µm. (B–K) *n* = 3; (L–O) *n* = 5. Data are presented as means ± SEM. Unpaired Student's *t*‐tests (B,D) were used to evaluate statistical significance. Data were analyzed using one‐way ANOVA (F,H,J,K,M,O) with the Tukey test.

To validate whether *Lkb1* deletion enhances PSC activation and CCL2 secretion via OSM in CD11c^+^CD206^+^ macrophages, *Lkb1*‐deleted CD11c^+^CD206^+^ macrophages were co‐cultured with PSCs (Figure 7A). *Lkb1* deletion significantly upregulated OSM mRNA expression in CD11c^+^CD206^+^ macrophages (Figure S14A), which was further corroborated by elevated OSM protein secretion as quantified by ELISA (Figure S14B). The *Lkb1*‐deleted CD11c^+^CD206^+^ macrophages induced marked PSC activation and CCL2 secretion, evidenced by significant increases in both mRNA and protein levels of α‐SMA and CCL2 (Figure 7B–E), whereas no marked difference was observed in CD11c^+^CD206^−^ cells (Figure S15A–C). To delineate the specific role of OSM, we treated PSCs with recombinant OSM. Dose‐response experiments demonstrated that increasing OSM concentrations potently upregulated CCL2 and α‐SMA expression (Figure 7F,G). Similarly, time‐course analysis using a fixed OSM concentration (50 ng/mL) revealed a concentration‐dependent induction of these profibrotic markers, confirming OSM's direct role in driving PSC activation and CCL2 secretion (Figure 7H,I). Notably, selective neutralization of OSM using a specific anti‐OSM antibody in the co‐culture system markedly attenuated PSC activation and *CCL2* expression (Figure 7J), establishing OSM as an essential mediator in this signaling cascade. To elucidate the underlying molecular mechanisms, PSCs were pretreated for 12 hours with an OSM‐neutralizing antibody, the MAPK pathway inhibitor PD98059, or the NF‐κB inhibitor curcumin prior to OSM stimulation. Both pharmacological inhibitors exhibited inhibitory efficacy comparable to OSM neutralization, indicating that OSM‐induced *CCL2* secretion in PSCs primarily occurs through the MAPK/NF‐κB signaling axis (Figure 7K).

To elucidate the downstream pathways mediating the upregulation of the inflammatory cytokine OSM following *Lkb1* deletion, we treated CD11c^+^CD206^+^ macrophages isolated from *Lkb1^f/f^* mice with pharmacological inhibitors targeting canonical *Lkb1* downstream kinases, including AMP‐activated protein kinase (AMPK), salt‐inducible kinase (SIK), microtubule affinity‐regulating kinase (MARK), and NUAK family SNF1‐like kinase 3 (NUAK3) [34, 35]. Analysis of OSM expression levels revealed that inhibition of SIK and MARK pathways resulted in significant OSM upregulation (Figure S16A), thereby identifying SIK and MARK as potential regulatory nodes in OSM secretion by CD11c^+^CD206^+^ macrophages. Meanwhile, therapeutic administration of an OSM‐neutralizing antibody prevented the *Lkb1* deletion‐induced increase in CCL2 secretion from PSCs and the infiltration of CD11c^+^CD206^+^ macrophages; this treatment also reduced collagen I and TGF‐β1 expression in pancreatic tissue, ultimately attenuating pancreatic inflammation and fibrosis (Figure 7L–O, Figure S16B–D). Collectively, these data demonstrate that *Lkb1* deletion drives OSM hypersecretion in CD11c^+^CD206^+^ macrophages, which subsequently engages OSMR on PSCs. This interaction activates MAPK and NF‐κB signaling cascades within PSCs, leading to enhanced secretion of CCL2 and accelerated ECM production. These molecular events collectively contribute to the exacerbation of CP‐associated fibrosis.

## 3 Discussion

Our study is the first to identify significant infiltration of CD11c^+^ cells accompanied by reduced *Lkb1* expression within fibrotic areas of clinical CP patients. Animal experiments confirmed that *Lkb1* deletion in CD11c^+^ cells promotes the infiltration of CD11c^+^CD206^+^ macrophages. In the fibrotic microenvironment of CP, these CD11c^+^CD206^+^ cells function as a mixed‐phenotype macrophage population with a distinct role, significantly exacerbating the progression of CP fibrosis. Comprehensive in vitro and in vivo functional analyses demonstrated that *Lkb1* deletion enhanced CCL2 secretion from CD11c^+^CD206^+^ macrophages via promoting STAT3 phosphorylation and facilitated the infiltration of this macrophage subset. In addition, CD11c^+^CD206^+^ macrophages remodel PSCs via OSM‐OSMR signaling, thereby facilitating the progression of fibrosis in CP. These findings provide important insights into the role of Lkb1 in the pathogenesis of CP and support the development of clinical interventions targeting CP‐associated fibrosis.

In clinical CP samples and animal models, a large number of CD11c^+^CD206^+^ macrophages were detected in pancreatic tissue. scRNA‐seq showed that *Lkb1* deletion could change the inflammatory microenvironment of CP, promote the differentiation of monocytes into CD11c^+^CD206^+^ macrophages, and drive the recruitment of macrophages characterized by the CD11c^+^CD206^+^ phenotype. These CD11c^+^CD206^+^ macrophages, distinct from classical M1 and M2 macrophages, exhibit characteristics of both M1 macrophages (marked by antigen‐presenting molecules and pro‐inflammatory cytokines) and M2 macrophages (characterized by genes associated with tissue maintenance and repair) [23, 36]. This suggests that the conventional macrophage classification system fails to fully capture the macrophage heterogeneity in CP. Moreover, CD11c^+^CD206^+^ macrophages have been identified in the fibrotic tissues of pancreatic cancer, where tumor‐derived factors induce their differentiation from monocytes, promoting the invasiveness of pancreatic cancer cells and advancing pancreatic cancer fibrosis and disease progression [37]. The secretion of pro‐inflammatory cytokines by macrophages is recognized as a significant driver of acinar‐to‐ductal metaplasia (ADM), a known precursor lesion to pancreatic cancer [38]. Our findings indicate that CD11c^+^CD206^+^ macrophages express high levels of classic pro‐inflammatory factors and chemokines. By promoting the ADM process, these macrophages likely help shape a tumor‐promoting microenvironment, thereby facilitating the malignant progression from chronic inflammation to pancreatic cancer. Our study revealed that CD11c^+^CD206^+^ macrophages not only secrete inflammatory factors but also directly activate PSCs, promoting ECM production. Recent studies have identified macrophage‐to‐myofibroblast transition (MMT) as a novel feature of CP. Although the present study did not directly validate the MMT phenomenon, the Mac‐Fib subpopulation identified by scRNA‐seq—characterized by high expression of ECM‐related genes such as Colla1and Col3a1—suggests that macrophages possess the potential to differentiate into fibroblast‐like cells. These findings imply that CD11c^+^CD206^+^ macrophages may also contribute directly to ECM production via the MMT pathway. These findings highlight the critical role of CD11c^+^CD206^+^ mixed‐phenotype macrophages in exacerbating fibrosis in CP due to *Lkb1* deletion, offering novel insights into the traditional M2 macrophage‐centric model of fibrogenesis in CP [27].

This study identified CCL2 as a key chemokine responsible for the enhanced recruitment of CD11c^+^CD206^+^ macrophages induced by *Lkb1* deletion. CCL2 is known to facilitate the recruitment and polarization of M2‐like macrophages [39] and plays a critical role in the pathogenesis of various chronic inflammatory diseases [40]. *Lkb1* deletion significantly upregulates CCL2 secretion in both CD11c^+^CD206^+^ macrophages and PSCs. Inhibition of the CCL2/CCR2 axis effectively reversed the enhanced infiltration of CD11c^+^CD206^+^ macrophages induced by *Lkb1* deletion, leading to a marked attenuation of both inflammatory severity and fibrotic progression in CP. In CD11c^+^CD206^+^ macrophage cells, STAT3 was identified as a key downstream TF of *Lkb1*. The STAT3 signaling pathway is implicated in inflammatory responses across multiple disease contexts [41, 42, 43], and its activation stimulates CCL2 secretion [44]. *Lkb1* deletion enhances CCL2 secretion by enhancing STAT3 phosphorylation in CD11c^+^CD206^+^ macrophages. Although *Lkb1* deletion in T cells has been previously shown to promote STAT3 activation [45], the current study demonstrates that *Lkb1* deletion in CD11c^+^ cells similarly induces STAT3 activation, indicating a conserved regulatory function of Lkb1. Previous studies have shown that OSM promotes inflammatory responses and fibroblast activation [46, 47]. Notably, Lee et al. recently demonstrated that macrophage‐derived OSM induces inflammatory gene expression in cancer‐associated fibroblasts (CAFs), thereby establishing a pro‐tumorigenic microenvironment in PDAC [48]. Furthermore, OSM enhances ECM collagen I fiber cross‐linking, facilitating cellular invasion [49]. In this study, we demonstrated that *Lkb1* deletion enhances OSM–OSMR signaling between CD11c^+^CD206^+^ macrophages and PSCs, activates the MAPK/NF‐κB pathway in PSCs, and subsequently upregulates CCL2 secretion. OSM neutralization effectively inhibited CCL2 secretion from PSCs induced by CD11c^+^CD206^+^ macrophages and alleviated the exacerbated fibrotic phenotype caused by *Lkb1* deletion. In summary, *Lkb1* deletion enhances CCL2 secretion from CD11c^+^CD206^+^ macrophages via STAT3 activation, leading to increased infiltration of these macrophages. Subsequently, CD11c^+^CD206^+^ macrophages further activate PSCs through OSM signaling and drive their differentiation into inflammatory fibroblasts. This cascade establishes a CCL2‐mediated positive feedback loop that recruits additional monocyte‐derived macrophages, ultimately promoting ECM production by PSCs and exacerbating fibrotic progression. These findings indicate that *Lkb1* deletion drives functional reprogramming of PSCs via OSM signaling, thereby modulating the inflammatory microenvironment and promoting the progression of CP fibrosis.

Therapeutic strategies targeting OSM blockade have advanced into clinical trials. In Phase I and II trials for rheumatoid arthritis, a humanized anti‐OSM monoclonal antibody (GSK315234) was evaluated [50]. Although clinical efficacy was modest, the drug demonstrated good tolerability with minimal adverse effects. Another anti‐OSM monoclonal antibody, GSK2330811, has advanced into clinical trials for systemic sclerosis [51]. Furthermore, CCL2/CCR2 antagonists have been investigated in clinical trials, confirming their safety in trials for liver fibrosis [52, 53] and showing promising therapeutic outcomes in patients with PDAC and severe fibrosis [54]. Both OSM and CCL2 have emerged as potential novel predictive biomarkers and therapeutic targets for CP, offering a promising strategy to address the current clinical challenge of suboptimal fibrosis treatment in CP, thus warranting further clinical investigations.

Although this study demonstrates several strengths, it also has certain limitations. In the absence of cerulein stimulation, *Lkb1* deletion did not significantly alter the population of Treg cells in pancreatic tissue [16]. In contrast, under CP conditions, *Lkb1* deletion led to enhanced infiltration of Treg cells. Inhibition of the CCL2/CCR2 axis attenuated Treg cell accumulation, indicating that this axis mediates the *Lkb1* deletion‐induced fibrosis in CP during inflammation. Nonetheless. The precise contribution of Treg cells to fibrotic processes and the underlying molecular mechanisms warrant further invest activated T cells. We acknowledge that our current experimental approach did not fully resolve the heterogeneity within this CD11c^+^ cellular compartment. Furthermore, we recognize that Lkb1 may exert functional roles in other immune cell populations within the pancreatic microenvironment, an important aspect that represents a significant direction for our future research investigations. Although obtaining pancreatic tissue samples from patients with early‐stage CP poses significant challenges in clinical practice, we established a refined animal model of CP to dynamically track temporal changes in Lkb1 expression within pancreatic CD11c^+^ cells at different modeling time points. Our findings reveal a progressive downregulation of Lkb1 expression in CD11c^+^ cells during CP progression. An initial population of CD11c^+^ cells with low Lkb1 expression secretes CCL2 to recruit circulating monocytes. These recruited monocytes are subsequently rapidly reprogrammed by the inflammatory microenvironment, leading to downregulation of Lkb1 and adoption of a pro‐fibrotic CD11c^+^CD206^+^ phenotype. Previous studies have reported that intraperitoneal administration of *E. coli* LPS in mice leads to a significant reduction in Lkb1 protein levels within CD11c^+^ cells, suggesting that an inflammatory microenvironment can downregulate Lkb1 expression [16]. However, the precise upstream mechanisms driving Lkb1 downregulation remain to be fully elucidated. Recent studies have shown that piericidin analogue S14, a novel marine‐derived compound, functions as an Lkb1 activator and significantly suppresses renal fibrosis [55]. Future studies will aim to elucidate the mechanisms underlying the downregulation of Lkb1 expression in CD11c^+^ cells within pancreatic tissues of patients with CP, and to evaluate the therapeutic potential of Lkb1 activators in mitigating CP‐associated fibrosis.

**FIGURE 8:**
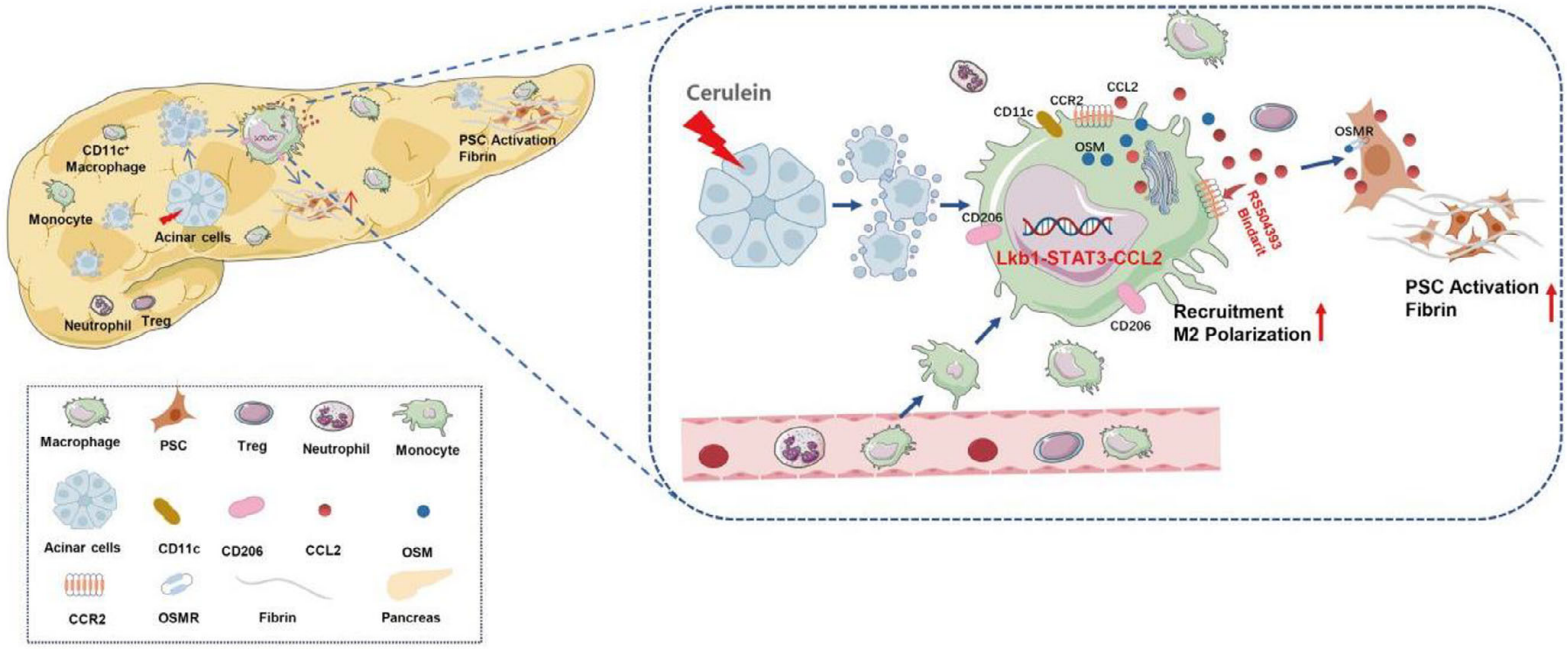
Mechanistic model of *Lkb1*’s role in CD11c^+^ cells in chronic pancreatitis.

This study uncovers a novel mechanism regulating ECM deposition and fibrosis progression in CP. Specifically, *Lkb1* deletion in CD11c^+^ cells enhances CCL2 secretion from CD11c^+^CD206^+^ macrophages, which in turn promotes macrophage infiltration. Via OSM signaling, these macrophages interact with PSCs, inducing functional reprogramming of PSCs and modifying the inflammatory microenvironment in the pancreas. These changes ultimately lead to exacerbated PSC activation and ECM accumulation (Figure 8). Clinical evidence further demonstrates reduced Lkb1 expression in CD11c^+^ cells from CP patients, along with positive correlations between OSM, CCL2 expression levels, and the extent of fibrosis. These findings highlight the therapeutic potential of targeting the OSM and CCL2/CCR2 axes in clinical strategies for CP.

## 4 Experimental Section

### 4.1 Animal Models

All the animals were male mice aged 6–8 weeks, with body weights of approximately 25 g. They were housed in specific pathogen‐free (SPF) barrier facilities and followed the protocols approved by the Animal Ethics Committee of the Affiliated Hospital of Qingdao University. *CD11c^Cre^* and *Lkb1^f/f^* mice were bred to generate *CD11c^Cre^Lkb1^f/f^* mice. CP was induced using repeated cerulein injections. *CD11c^Cre^Lkb1^f/f^* and *Lkb1^f/f^* mice received intraperitoneal injections of cerulein (100 µg/kg) every hour for 6 h, three times a week, for 3 weeks. Three days after the final injection, the mice were euthanized, and the pancreatic tissues were harvested. For the binding CCL2 inhibitor experiment, mice were administered Bindarit 50 mg/kg/day for 3 days per week for 3 consecutive weeks. In the RS504393 CCR2 inhibitor experiment, mice were administered 4 mg/kg/day for 3 days per week for 3 consecutive weeks. In the OSM and antibody experiments, mice received anti‐OSM (AF‐495‐NA) at 1 mg/kg/day for 3 days per week over a 3‐week period. Pancreatic tissues were collected 3 days after the final injection—ethical code (AHQU‐MAL20201231ZWQ).

### 4.2 scRNA‐seq

Transcriptomic analysis was performed using 3′ transcriptome technology from 10× Genomics (GENE DENOVO, Guangzhou, China), enabling simultaneous expression profiling of 500 to 10 000 cells from each sample via short‐read sequencing and microfluidic technology. Sequencing libraries were constructed and subjected to high‐throughput paired‐end sequencing on an Illumina NovaSeq Plus platform. Official Cell Ranger software (10× Genomics) was used for raw data quality control and alignment with the reference genome. After alignment, Cell Ranger filtered and corrected barcodes and unique molecular identifiers, followed by cell validation and filtering. The resulting scRNA‐seq data were further processed and analyzed using the R package Seurat.

### 4.3 Pseudo‐Time Trajectory Analysis

Monocle2 organizes individual cells along a pseudo‐time axis by analyzing the critical gene expression patterns. This approach captures asynchronous biological processes at the single‐cell level, mapping cells along a trajectory indicative of developmental events such as cell differentiation. The data were projected onto a two‐dimensional plane by inputting the gene expression matrix into Monocle2, resulting in a tree‐like differentiation trajectory with distinct branches and nodes. The earliest cell population, marked by the lowest pseudo‐time value, served as a reference for calculating the pseudo‐time across all cells.

Monocle2 further classified cell segments into distinct differentiation states and identified differentially expressed genes with a false discovery rate of less than 1e‐5. It models gene expression as a smooth, nonlinear function of pseudo‐time to determine correlations with changes in expression, with qualifying genes marked as differentially expressed.

### 4.4 Data‐Independent Acquisition (DIA) Experiments and Analytical Methods

Pancreatic tissues from mouse CP were isolated using magnetic beads to collect CD11c^+^ cell samples. The iST sample pre‐processing kit was utilized to assist in the extraction, denaturation, reduction, alkylation, enzymatic digestion, and desalting of proteins from the samples, ultimately allowing for the peptide samples to be preserved at −80°C. Subsequently, a reference spectral library was created using high‐pH reverse‐phase separation, data‐dependent acquisition, and database searching. The peptide samples were analyzed using an LC‐MS/MS system, where they were introduced into a mass spectrometer and analyzed using DIA.

Various annotation techniques, including Gene Ontology (GO), Kyoto Encyclopedia of Genes and Genomes (KEGG), and COG/KOG, were used to analyze the molecular functions of the proteins. Advanced methods, such as protein domain, transcription factor, and subcellular localization analyses, provide insights into protein characteristics and intracellular roles. Statistical methods such as PCA (principal component analysis) and correlation heatmaps reveal data relationships. Volcano plots, heat maps, and enrichment analyses (GO, KEGG, Orthology, Disease Ontology, and Reactome pathways) were used to assess differential protein expression and biological relevance. Additionally, protein–protein interaction network analysis and Gene Set Enrichment Analysis (GSEA) were used to explore protein interactions and gene expression trends in specific biological processes.

### 4.5 Cellular Communication Analysis (CellChat)

CellChat V2.1.0 utilizes single‐cell gene expression matrices to analyze ligand‐receptor interactions within specific cellular subpopulations. It constructs ligand‐receptor interaction networks to predict intercellular communication pathways and identify significant interactions through expression abundance and *p*‐values. This approach highlights critical communication pairs and examines their expression levels to identify genes that influence the interactions between key cell types. The software quantifies the communication probabilities of ligand‐receptor pairs related to various signaling pathways, facilitating cross‐comparisons among different subpopulations. Additionally, it evaluates the effects of experimental treatments on intercellular communication by comparing ligand‐receptor interactions across samples to reveal enhanced signaling patterns due to interventions.

### 4.6 Weighted Gene Co‐Expression Network Analysis (WGCNA)

After gene filtering, the gene expression values were imported into WGCNA, and co‐expression modules were constructed using the automatic network construction function blockwise modules. The node and edge files of each module were imported into the Cytoscape software to generate the regulatory network diagram, with the transcription factor labelled in the diagram.

### 4.7 RNA Sequencing and Data Analysis

Total RNA was extracted from the pancreatic tissue of mouse CP or CD11c magnetic bead‐sorted cells (Miltenyi Biotec, #130‐125‐835) using TRIzol reagent, following the manufacturer's guidelines. RNA purification, reverse transcription, library preparation, and sequencing were conducted at Shanghai Majorbio Bio‐pharm Biotechnology Co., Ltd. (Shanghai, China), following the manufacturer's protocols (Illumina, San Diego, CA, USA). The expression levels of each transcript were calculated to identify differentially expressed genes between the two samples using the transcripts per million reads method. GO functional enrichment and KEGG pathway analyses were performed using Goatools and Python SciPy libraries, respectively.

### 4.8 Histology and Morphometric Analysis

Pancreatic tissues from mice were fixed in 10% formalin, then embedded in paraffin and sectioned into 4 µm slices for haematoxylin and eosin staining and Masson staining. Images were randomly acquired following scanning with a slide scanner (Axioscan7, ZEISS).

In the assessment of CP pancreatic sections, the classification of abnormal pancreatic tissue structure was as follows: 0, none; 1, rare; 2, mild (<10%); 3, moderate (10%–50%); and 4, severe (>50%). Glandular atrophy within these regions was graded as follows: 0, none; 1, mild (<10%); 2, moderate (10%–50%); and 3, severe (>50%). The presence of immune cells was categorized as follows: 0, none; 1, very few; 2, mild; 3, moderate; and 4, severe. The total histological score comprised the evaluation of the pancreatic tissue structure, glandular atrophy, and the presence of immune cells. The Masson staining score was determined based on the percentage of collagen fibers present.

In the assessment of acute pancreatitis (AP) pancreatic sections, pancreatic oedema was defined as follows: 0, none; 1, expansion of interlobular septae; 2, expansion of interlobular septae; 3, expansion of interacinar septae; 4, expansion of intercellular septae. Inflammatory cell infiltration: 0, none; 1, ducts (around ductal margins); 2, parenchyma (<33% of the lobules); 3, parenchyma (>33% and 67% of the lobules). For acinar cell death: 0, none; 1, focal acinar cell death (<5%); 2, focal acinar cell death (5%–20%); 3, diffuse acinar cell death (20%–50%); and 4, diffuse acinar cell death (>50%). The total histological score included pancreatitis oedema, acinar cell death, and the presence of inflammatory cells.

### 4.9 IHC and Immunofluorescence Staining

In brief, the tissue sections were incubated at 60°C for 2 h, then immersed in xylene and graded with ethanol. Endogenous peroxidase activity was inhibited using hydrogen peroxide, and antigen retrieval was performed with citrate buffer (pH 6.0). The sections were blocked with a 10% goat serum blocking solution (ZSGB‐BIO, #ZLI‐9021) at 37°C for 30 min. The primary antibody was applied and incubated overnight at 4°C. Subsequently, the mouse/rabbit universal secondary antibody (ZSGB‐BIO, #PV‐9000) was incubated at 37°C for 30 min. After that, DAB (ZSGB‐BIO, #ZLI‐9018) staining was performed, followed by hematoxylin (Solarbio, #G1140) staining. After dehydration and sealing with neutral balsam, the samples were examined under a microscope (Leica).

A four‐color multiple fluorescent immunohistochemical staining kit was used for co‐staining the mouse pancreatic tissue (Absinbio, #abs50028). The ALphaTSA multiplex IHC kit for tissues was used for co‐staining human pancreatic tissue (#AXT36100031; Alphaxbio). The concentrations of all primary antibodies matched the concentrations specified for immunohistochemistry, as previously described. The tissue sections were incubated with rabbit/mouse horseradish peroxidase‐conjugated secondary antibody for 30 min at 37°C. Subsequently, DAPI working solution (1×) was applied to cover the sample area and was incubated for 5 min at 37°C. Images were randomly acquired following scanning with a slide scanner (Axioscan7, ZEISS). Antibodies used in this study are listed in Table S6.

### 4.10 Flow Cytometry Analysis

The pancreatic tissue was enzymatically digested using collagenase type IV (2 mg/mL, Worthington, #LS004188) to generate a single‐cell suspension. Single‐cell suspensions were stained with various antibodies (Table S7). Data acquisition was performed using a BD Celesta instrument, and subsequent analysis was performed using FlowJo software (San Carlos). Flow cytometry was conducted on a FACSCanto flow cytometer, and the resulting data were analyzed using FlowJo software (v10.8).

### 4.11 RT‐qPCR

Total RNA was extracted from pancreatic tissues using a Total RNA Purification Kit (Accurate Biotechnology, #AG21101). cDNA was synthesized following the manufacturer's protocol using a One Scroll cDNA Synthesis Kit (Accurate Biotechnology, #AG11711). For RT‐qPCR, SYBR Green Pro Taq HS Premix (Accurate Biotechnology, #AG11701) and a CFX96TM Real‐Time System (Bio‐Rad, CA, USA) were used. Primer sequences are provided in Table S8. All primers were purchased from BGI Tech Solutions (Beijing Liuhe) Co.

### 4.12 Isolation and Culture of Primary CD11c^+^ CD206^+^ Cells

Bone marrow cells were isolated from *CD11c^Cre^Lkb1^f/f^* and *Lkb1^f/f^* mice under sterile conditions. After extraction, the cells underwent screening, red blood cell lysis, and washing to obtain bone marrow‐derived monocytes. These monocytes were cultured in RPMI medium supplemented with granulocyte‐macrophage colony‐stimulating factor (GM‐CSF) (20 ng/mL, Peprotech, #315‐03). A total of 2 × 10^6^ CD11c^+^ cells were seeded in a 60 mm dish. On day 6 of culture, LPS (1 µg/mL, Sigma‐Aldrich, #L2630) and IL‐4 (20 ng/mL, Protech, #214‐14) were added, and CD11c^+^CD206^+^ cells were sorted by flow cytometry. LPS (1 µg/mL, Sigma‐Aldrich, #L2630) was added to induce an inflammatory response, while the inhibitor group received Static (10 µM, MCE, #HY13818). After 12 h of culture, the CD11c^+^ CD206^+^cells were harvested for RT‐qPCR or Western blot analysis.

### 4.13 Co‐Culture of Primary CD11c^+^CD206^+^ Cells and CD11c^+^CD206^−^ Cells With PSCs

Mouse PSCs, generously provided by Professor Jing Xue from Shanghai Jiao Tong University School of Medicine, were used for experiments after the third passage. Primary CD11c^+^CD206^+^ cells were isolated and cultured from the bone marrow of *CD11c^Cre^Lkb1^f/f^* and *Lkb1^f/f^* mice as previously described. For co‐culture, PSCs (2 × 10^5^ cells) were seeded in the lower chamber of a 6‐well Transwell plate, while LPS‐stimulated CD11c^+^ *CD206^+^* cells and CD11c^+^CD206^−^ cells (2 × 10^6^ cells) were placed in the upper chamber (pore size, 3.0 µm). After 24 h of co‐culture, PSCs were harvested for further analysis. For the neutralizing antibody experiment, OSM‐neutralizing antibody (1 µg/mL) was applied directly during LPS stimulation. After a 12‐h pre‐incubation, the mixture was co‐cultured with PSCs.

### 4.14 Immunofluorescence Staining of Cells

For primary CD11c^+^CD206^+^ cells and co‐culture experiments, cells were cultured on cell‐climbing slices. Cells were fixed with 4% paraformaldehyde at room temperature. Endogenous peroxidase activity was blocked using 3% H_2_O_2_, and cells were permeabilized with PBS containing 0.25% Triton X‐100 for 10 minutes at room temperature. After blocking with 5% serum for 30 min, cells were incubated with primary antibodies overnight at 4°C. HRP‐conjugated secondary antibodies were applied for 30 min at room temperature, followed by PBS washes. Signal amplification was achieved using a freshly prepared 1 × TSA working solution for 10 min. For multiplex staining, antibodies were eluted with preheated stripping buffer, followed by re‐blocking and additional rounds of staining for other targets. Nuclei were counterstained with DAPI (10 min, RT), and coverslips were mounted with antifade medium. Images were acquired using a fluorescence microscope and analyzed.

### 4.15 Enzyme‐Linked Immunosorbent Assay (ELISA)

On day 6 of culture, CD11c^+^CD206^+^ cells were harvested and centrifuged, and the supernatants were collected. The protein levels of CCL2 and OSM were quantified using mouse ELISA kits (RUIXIN BIOTECH, #RXW201283M, #RXW202195M).

### 4.16 Western Blot Analysis

Proteins extracted from CD11c^+^ CD206^+^ cells and CD11c^+^CD206^−^ cells were subjected to electrophoresis on a 10% Tris SwePAGE gel (Servicebio, #G2302‐10) and transferred onto a PVDF membrane. After blocking with 5% milk, the membranes were immunoblotted using the following antibodies: Lkb1 (1:1000; Cell Signaling, #3047), STAT3 (1:1000; Cell Signaling, #12 640), p‐STAT3 (1:1000; Cell Signaling, #9134), JAK2 (1:1000; Cell Signaling, #3230), p‐JAK2 (1:1000; Cell Signaling, #3776), CCL2 (1:1000; Abcam, #ab25124), α‐SMA(1:1000; Abcam, ab5694) and GAPDH (1:1000; Aksomics, #KC‐5G4). The immunoblotted membrane was then incubated with HRP‐conjugated secondary antibodies for 1 h at room temperature. Finally, the signals from the immunoblotting were detected using a fluorescent immunoblotting substrate, and digital images were captured using a Touch Imager XLi (e‐BLOT).

### 4.17 Human Specimens

The Shanghai Changhai Hospital generously provided pancreatic tissue samples from patients diagnosed with CP, whereas normal pancreatic tissue samples were obtained from the Affiliated Hospital of Qingdao University. Patients with CP present with clinical manifestations such as chronic or recurrent abdominal pain, with diagnoses confirmed using computed tomography and ultrasound imaging. Normal pancreatic specimens were obtained from organ transplant donors with healthy pancreatic tissue. Informed consent was obtained from all participants, and the study was approved by the Research Ethics Committee of the Affiliated Hospital of Qingdao University. Ethical code (QYFYWZLL29083).

### 4.18 Statistical Analysis

The Prism GraphPad7 software was used for statistical analysis. All data are expressed as mean ± SEM. Two‐tailed Student's *t*‐tests were used for comparisons between two groups, while one‐way analysis of variance (ANOVA) was used for multi‐group comparisons, followed by Tukey's multiple comparisons test. *p*‐values <0.05 were considered statistically significant.

## Author Contributions

Conceptualization: Wenqing Zhang, Shan Guo, and Yu Zhang. Methodology: Wenqing Zhang, Shan Guo, Yu Zhang, and Hongqing Luo. Investigation: Ke Lei and Chenyang Zhao. Visualization: Qian Yu and Yujing Xiao. Funding acquisition: Xiaoyu Li and He Ren. Project administration: Ke Lei, Chenyang Zhao, and Qian Yu. Supervision: Xiaoyu Li, He Ren, and Xiaoming Feng. Writing – original draft: Wenqing Zhang, Shan Guo, and Yu Zhang. Writing – review & editing: Xiaoyu Li, He Ren, and Xiaoming Feng.

## Funding

Natural Science Foundation of China (82270676, XYL); National Science Fund for Distinguished Young Scholars (82125026, HR); Key Program of National Natural Science Foundation of China (82330081, HR); Taishan Young Scholars Program of Shandong Province (tsqn202306395, XYL); 2023 Qingdao Technology Benefiting Demonstration Project (23‐2‐8‐smjk‐8‐nsh, XYL); “Medicine Plus ” Joint Research Program of Qingdao University (YX2024201, HR).

## Conflicts of Interest

The authors declare no conflicts of interest.

## Supporting information




**Supporting File 1**: advs75018‐sup‐0001‐SuppMat.docx.

**Supporting File 2**: advs75018‐sup‐0002‐TableS1.xlsx.

**Supporting File 3**: advs75018‐sup‐0003‐TableS2.xlsx.

**Supporting File 4**: advs75018‐sup‐0004‐TableS3.xlsx.

**Supporting File 5**: advs75018‐sup‐0005‐TableS4.xlsx.

**Supporting File 6**: advs75018‐sup‐0006‐TableS5.xlsx.

**Supporting File 7**: advs75018‐sup‐0007‐TableS6.xlsx.

**Supporting File 8**: advs75018‐sup‐0008‐TableS7.xlsx.

**Supporting File 9**: advs75018‐sup‐0009‐TableS8.xlsx.

## Data Availability

The data that supports the findings of this study are available in the supplementary material of this article.
