# Supplementary material for: Liver Kinase B1 in CD11c+ Cells Inhibits Fibrosis in Chronic Pancreatitis via the Oncostatin M Signaling
Source: Adv Sci (Weinh). 2026 Mar 30;13(32):e75018. doi: 10.1002/advs.75018 (PMC13252620; doi:10.1002/advs.75018)
Supplement: Supplementary file 1 — Supporting File 1: advs75018‐sup‐0001‐SuppMat.docx. [file ADVS-13-e75018-s001.docx]

Supporting Information for

Liver kinase B1 in CD11c^+^ cells inhibits fibrosis in chronic pancreatitis via the Oncostatin M signaling

*Wenqing Zhang†, Shan Guo†, Yu Zhang†, He Ren*, Ke Lei, Chenyang Zhao, Qian Yu, Hongqing Luo, Yujing Xiao, Xiaoming Feng*, and Xiaoyu Li**

This file includes：

Figure S1 to S16


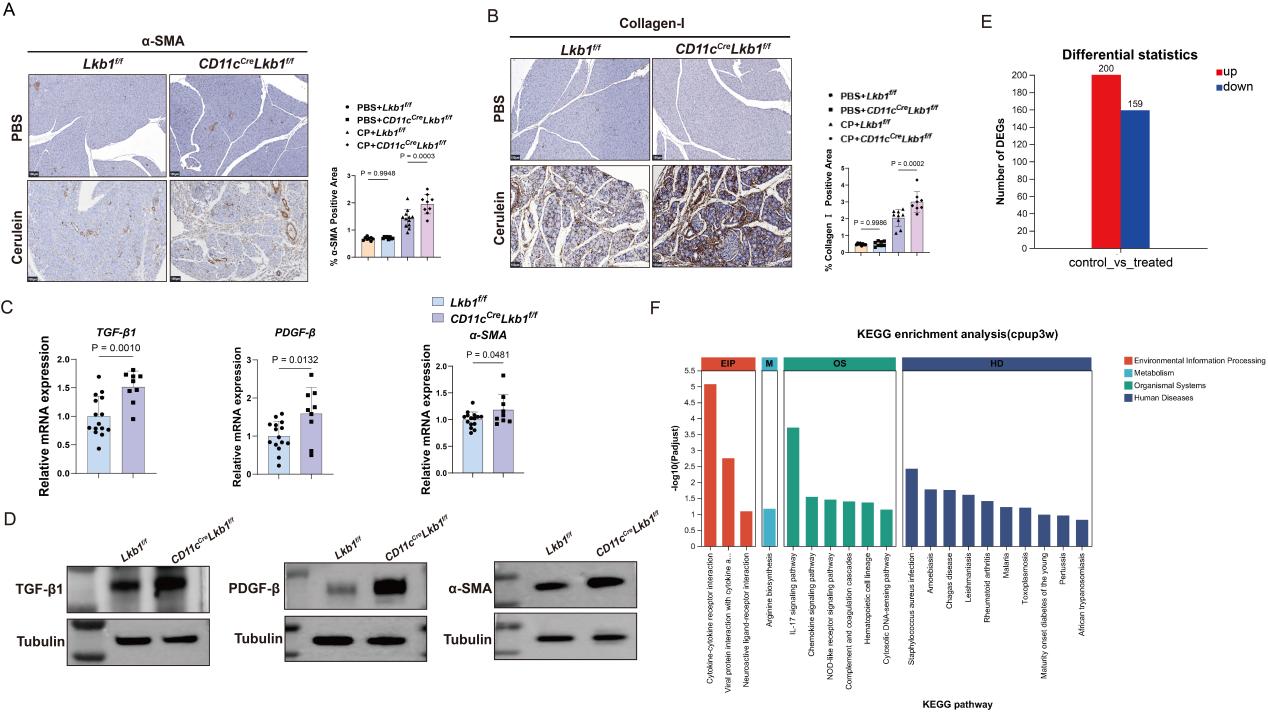


**Figure S1.** *Lkb1* deletion in CD11c^+^ cells promotes PSC activation and enhances inflammatory signaling pathways in CP pancreatic tissue.

**(A, B)** Representative IHC staining images of α-SMA and collagen-I. Scale bar, 100 µm. From left to right: *n* = 8; *n* = 8; *n* = 12; *n* = 10. **(C)** mRNA expression levels of *TGF-β1*, *PDGF-β,* and *α-SMA* in pancreatic tissue of *Lkb1^f/f^* (*n* = 15) and *CD11c^Cre^Lkb1^f/f^* (*n* = 9) mice. **(D)** Protein expression levels of *TGF-β1*, *PDGF-β,* and *α-SMA* in pancreatic tissue of *Lkb1^f/f^* (*n* = 3) and *CD11c^Cre^Lkb1^f/f^* (*n* = 3) mice. **(E)** Statistical analysis of differential expression in CP transcriptomic sequencing. **(F)** KEGG enrichment analysis results of upregulated genes from CP transcriptomic sequencing. (E, F) *n* = 4. Unpaired Student’s t tests (C) were used to evaluate statistical signiﬁcance. Data were analyzed using one-way ANOVA (A, B) with the Tukey test.

**
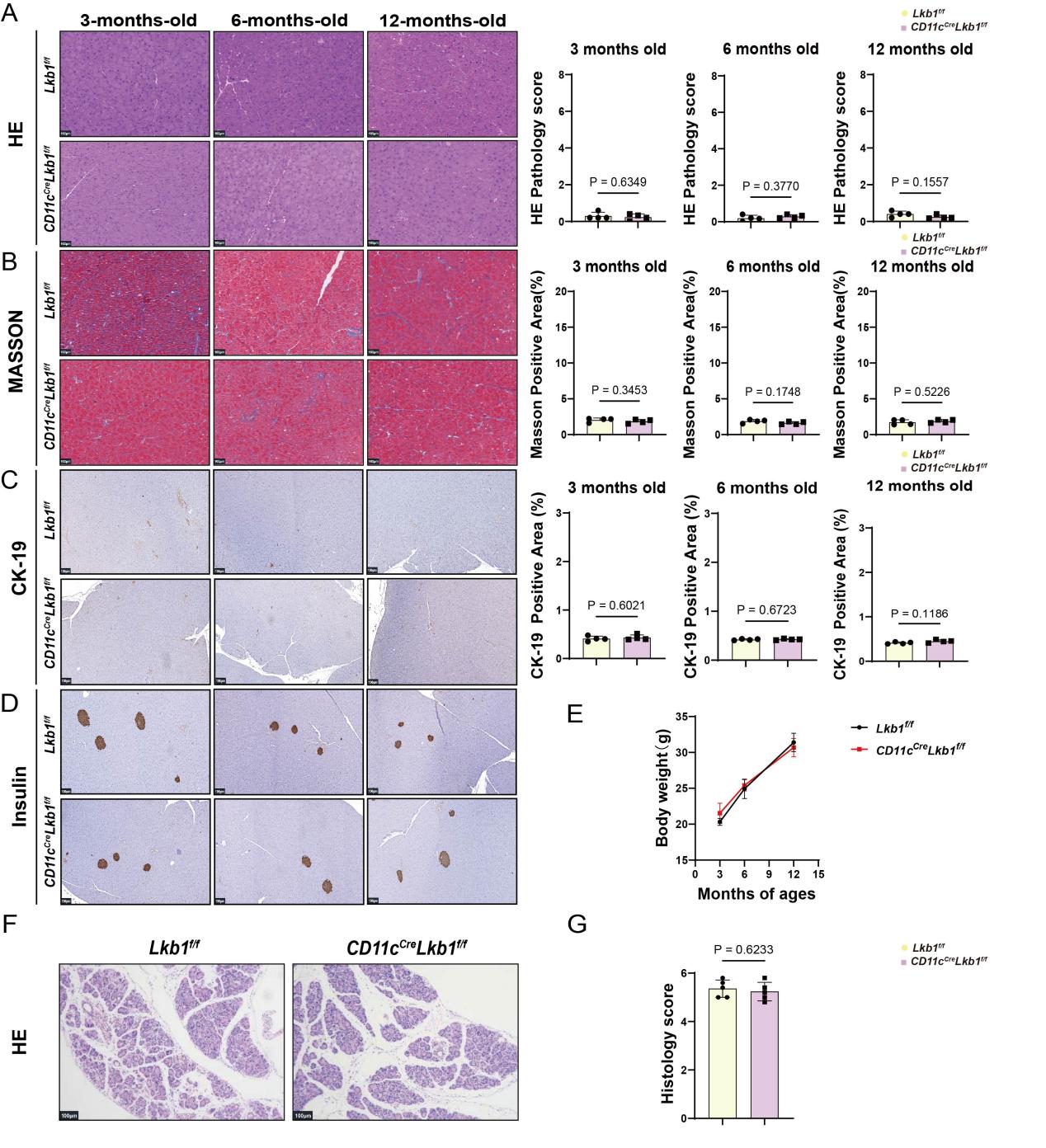
**

**Figure S2.** Histological characteristics of the pancreas in *Lkb1^f/f^* and *CD11c^Cre^Lkb1^f/f^* mice at different ages in an acute pancreatitis (AP) model.

**(A)** Representative images of pancreatic H&E staining. Scale bar, 100 µm. **(B)** Representative images of pancreatic Masson's staining. Scale bar, 100 µm. **(C)** Representative IHC staining images of CK19. Scale bar, 100 µm. **(D)** Representative IHC staining images of insulin. Scale bar, 100 µm. **(E)** Changes in body weight of mice at different ages. **(F, G)** Pancreatic H&E staining. Scale bar, 100 µm. (A-E) *n* = 4; (F, G) *n* = 5. Data are means ± SEM. Unpaired Student’s t tests were used to evaluate statistical signiﬁcance.


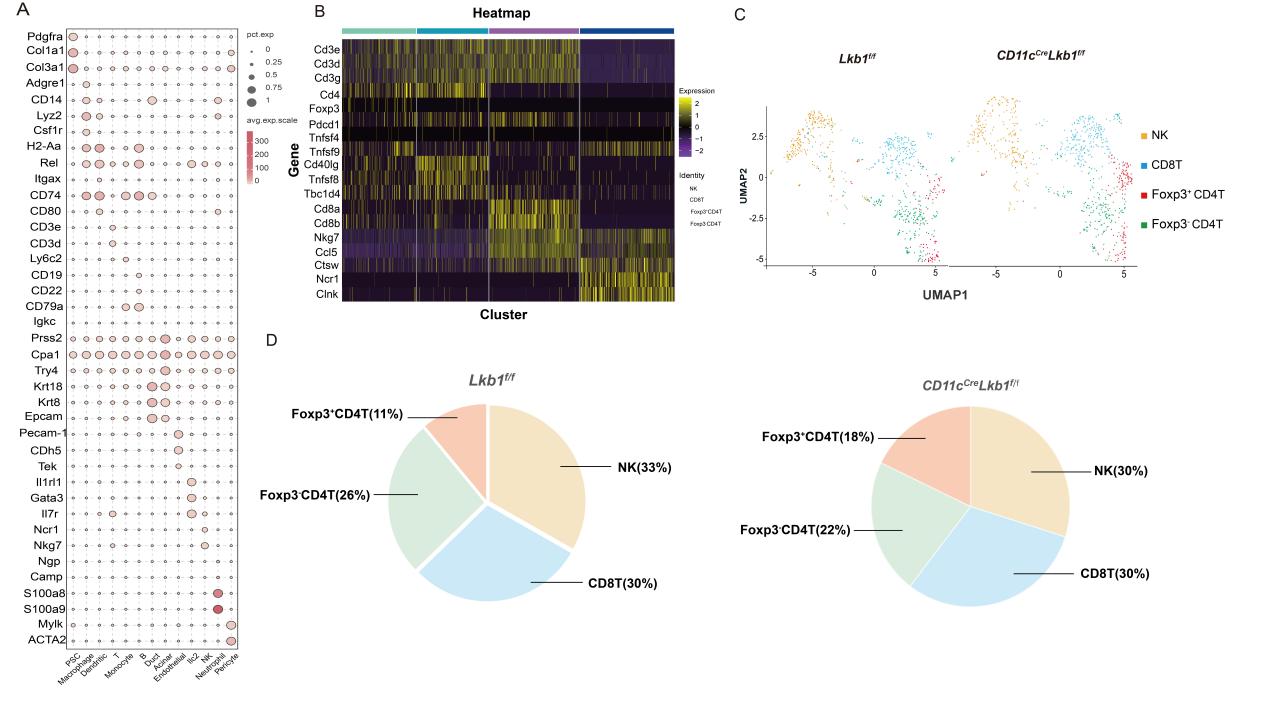


**Figure S3.** Differential genes marking different types of cells in scRNA-seq and deletion of *Lkb1* in CD11c^+^ cells alters the T cell phenotype in CP.

**(A)** Dot plot of the mean expression of canonical marker genes for the 13 major lineages as indicated. **(B)** Heatmap showing differentially expressed genes serving as different T cell subset phenotypic markers. **(C)** UMAP plot of T cells subclusters in CP pancreatic tissue. **(D)** Percentage of cells of different T cell subclusters in T cells of *Lkb1^f/f^* and *CD11c^Cre^Lkb1^f/f^* mice. (A-D) *Lkb1^f/f^* (*n* = 4) and *CD11c^Cre^Lkb1^f/f^* (*n* = 3) mice.


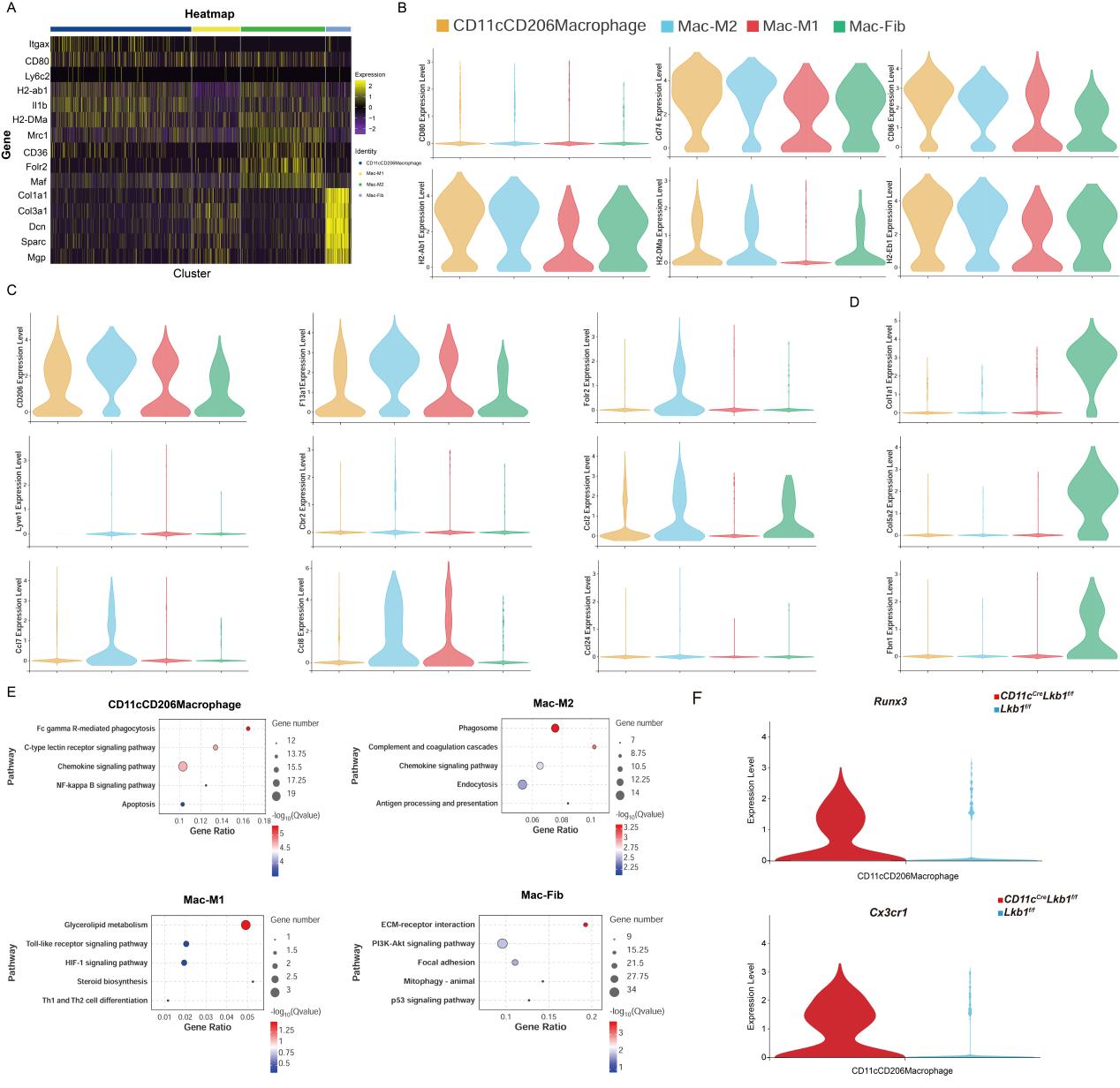


**Figure S4.** Definition and function of macrophage subsets in pancreatic tissue of *Lkb1^f/f^* and *CD11c^Cre^Lkb1^f/f^* mice.

**(A)** Heatmap showing differentially expressed genes serving as different macrophage subset phenotypic markers. **(B-D)** Violin plots of the gene expression of cell identity genes in various macrophage subsets. **(E)** KEGG enrichment analysis of upregulated differentially expressed genes in various macrophage subsets. **(F)** The violin plot shows the expression levels of *Runx3* and *Cx3cr1* in CD11c^+^CD206^+^ macrophages. (A-F) *Lkb1^f/f^* (*n* = 4) and *CD11c^Cre^Lkb1^f/f^* (*n* = 3) mice.


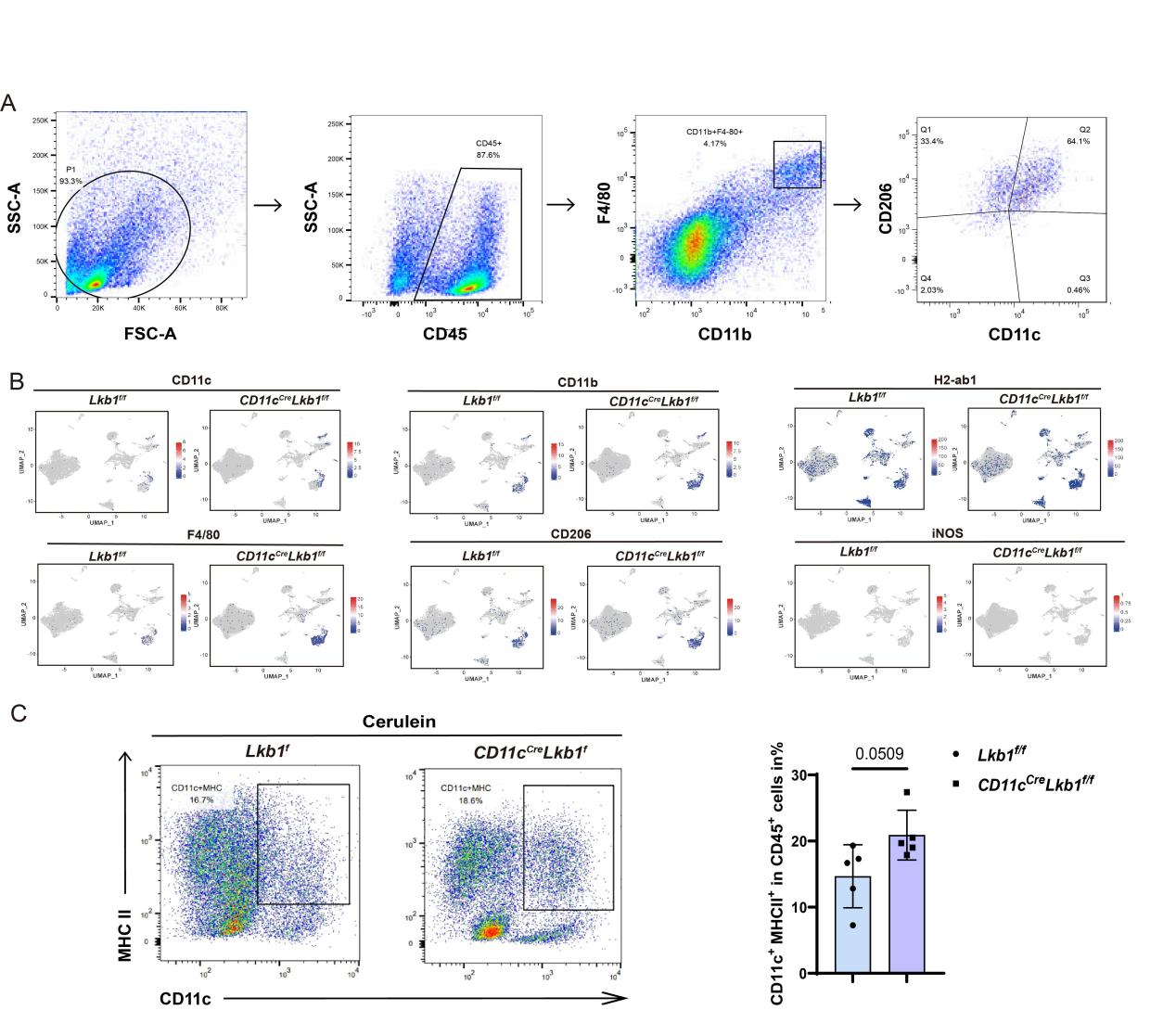


**Figure S5.** *Lkb1* deletion in CD11c^+^ cells promotes the expansion of CD11c^+^CD206^+^ macrophages, while exhibiting no significant impact on CD11c^+^ dendritic cells.

**(A)** Flow cytometry gating strategy. **(B)** UMAP display of gene expression of the indicated gene. *Lkb1^f/f^* (*n* = 4) and *CD11c^Cre^Lkb1^f/f^* (*n* = 3) mice. **(C)** Representative flow cytometry plots and percentage of CD11c^+^MHCII^+^ cells in pancreatic tissue. *n* = 5. Data are means ± SEM. Unpaired Student’s t tests were used to evaluate statistical signiﬁcance.


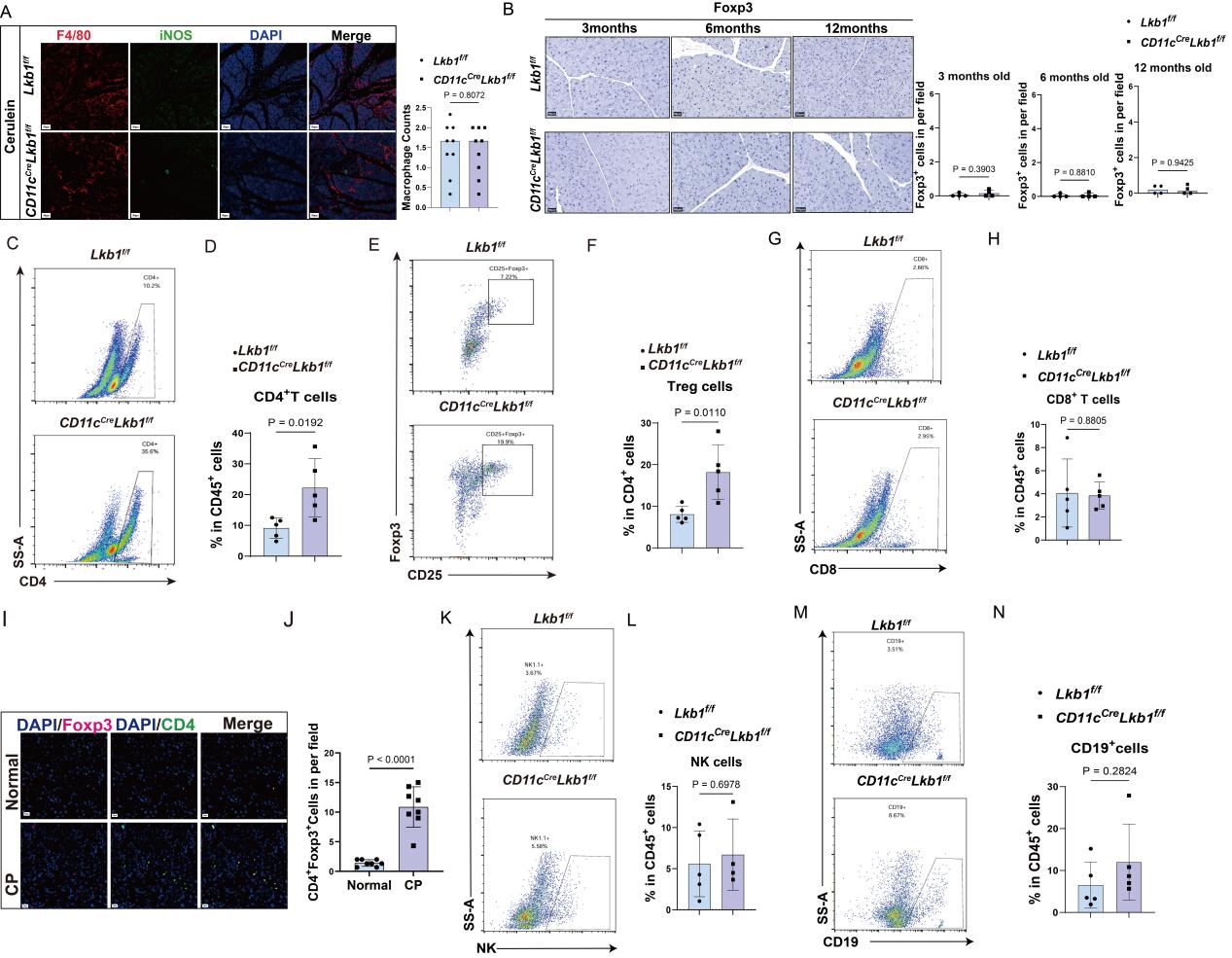


**Figure S6.** The effect of *Lkb1* deletion in CD11c^+^ cells on the infiltration of different immune cell populations.

**(A)** Representative immunofluorescence images of co-staining for F4/80, iNOS, and DAPI in the pancreas. Scale bar, 50 µm. *n* = 9. **(B)** Representative IHC staining images of Foxp3. Scale bar, 50 µm. *n* = 4. **(C, D)** Representative flow cytometry plots and percentages of CD4^+^ T cells in the pancreas. *n* = 5. **(E, F)** Representative flow cytometry plots and percentages of Treg cells in the pancreas. *n* = 5. **(G, H)** Representative flow cytometry plots of CD8^+^ cells in pancreatic macrophages from different groups of mice with CP. *n* = 5. **(I, J)** Representative immunofluorescence images of CD4, Foxp3 and DAPI co-staining in pancreatic tissues of patients with clinical CP. *n* = 8. **(K, L)** Representative flow cytometry plots of NK cells in pancreatic macrophages from *Lkb1^f/f^* (*n* = 5) and *CD11c^Cre^Lkb1^f/f^* (*n* = 4) mice with CP. **(M, N)** Representative flow cytometry plots of CD19^+^ cells in pancreatic macrophages from different groups of mice with CP. *n* = 5. Data are means ± SEM. Unpaired Student’s t tests were used to evaluate statistical signiﬁcance.

**
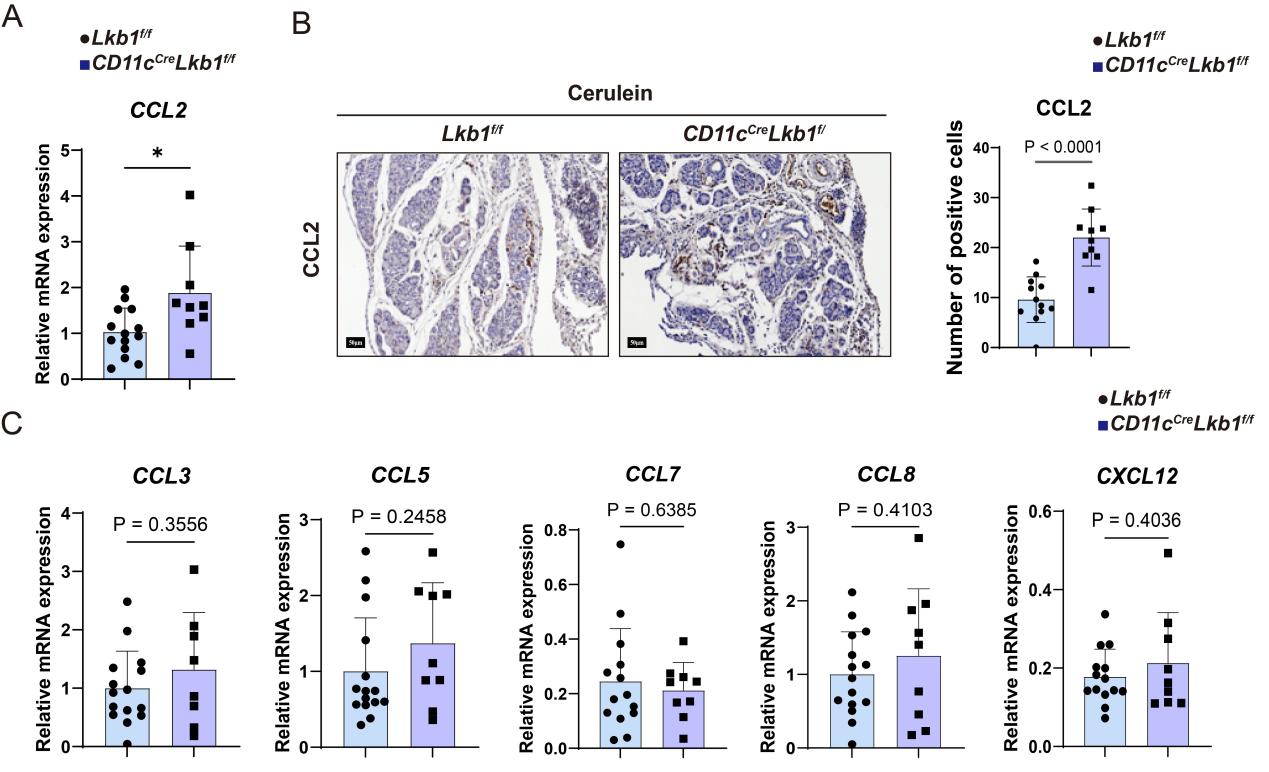
**

**Figure S7.** *Lkb1* deletion in CD11c^+^ cells does not affect the expression of other monocyte/macrophage chemokines in the CP mouse model.

**(A)** mRNA expression level of *CCL2* in CP pancreatic tissue. From left to right: *n* = 15; *n* = 9. **(B)** Representative IHC staining images of CCL2 in CP pancreatic tissue with statistical analysis. Scale bar, 50 µm. From left to right: *n* = 12; *n* = 10. **(C)** mRNA expression levels of *CCL3* (*n* = 15, *n* = 8), *CCL5* (*n* = 15, *n* = 8), *CCL7* (*n* = 14, *n* = 9), *CCL8* (*n* = 15, *n* = 9)*,* and *CXCL12* (*n* = 14, *n* = 9) in pancreatic tissue. Data are means ± SEM. Unpaired Student’s t tests were used to evaluate statistical signiﬁcance.


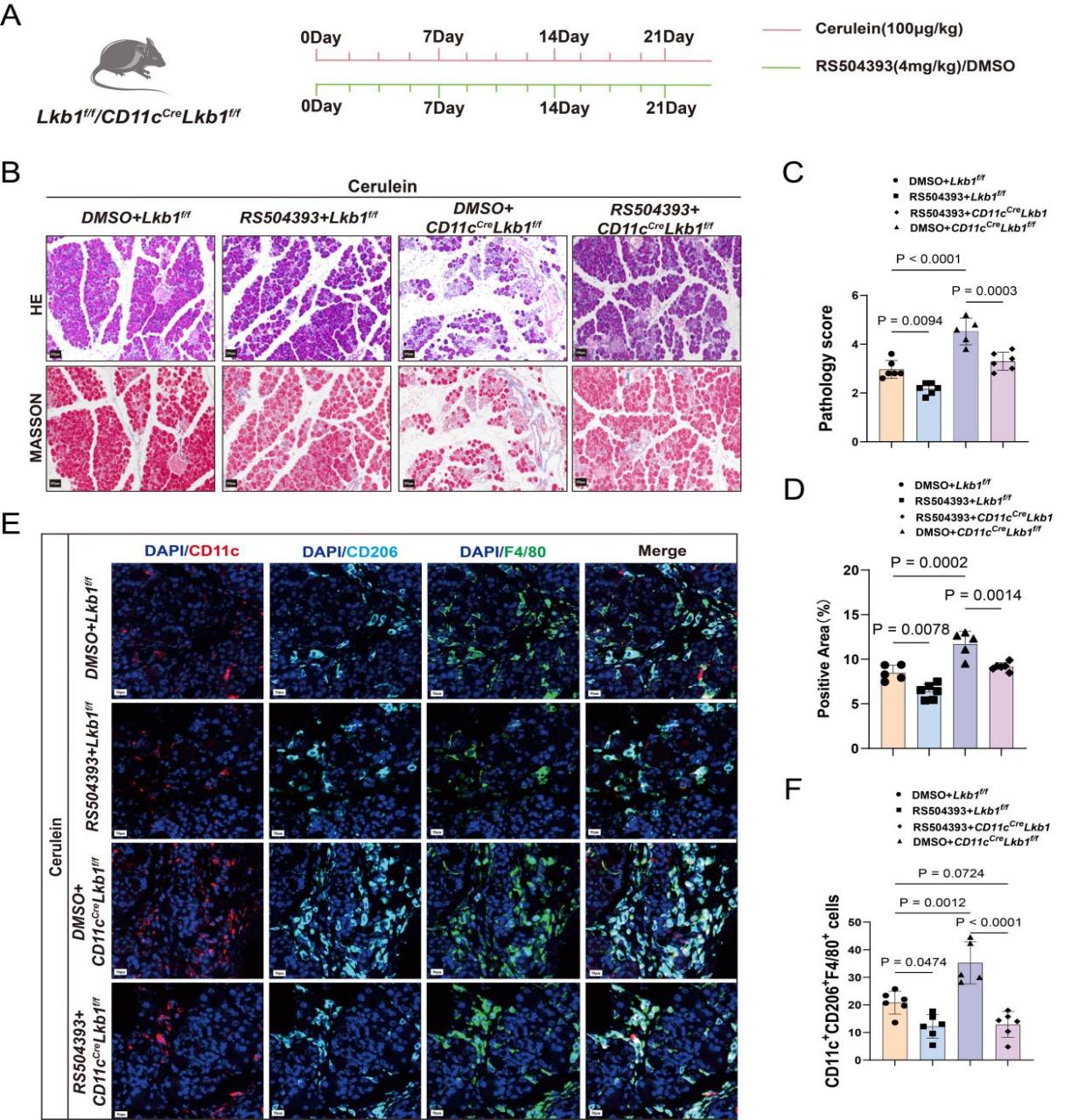


**Figure S8.** *Lkb1* deletion in CD11c^+^ cells promotes inflammation and fibrosis in CP through the CCL2/CCR2 axis.

**(A)** Schematic representation of the experimental model using CCR2 inhibitors. **(B)** Representative images of pancreatic tissue stained with H&E (*n* = 6; *n* = 6; *n* = 5; *n* = 6) staining and Masson's (*n* = 5; *n* = 6; *n* = 5; *n* = 6) staining with **(C, D)** pathological scores and statistical analysis. Scale bar, 100 µm. **(E)** Representative immunofluorescence images of CD11c, F4/80, CD206, and DAPI co-staining in pancreatic tissue with **(F)** statistical analysis. Scale bar, 50μm. From left to right: *n* = 6; *n* = 6; *n* = 5; *n* = 6. Scale bar, 50 μm. Data are means ± SEM. Data were analyzed using one-way ANOVA with the Tukey test.


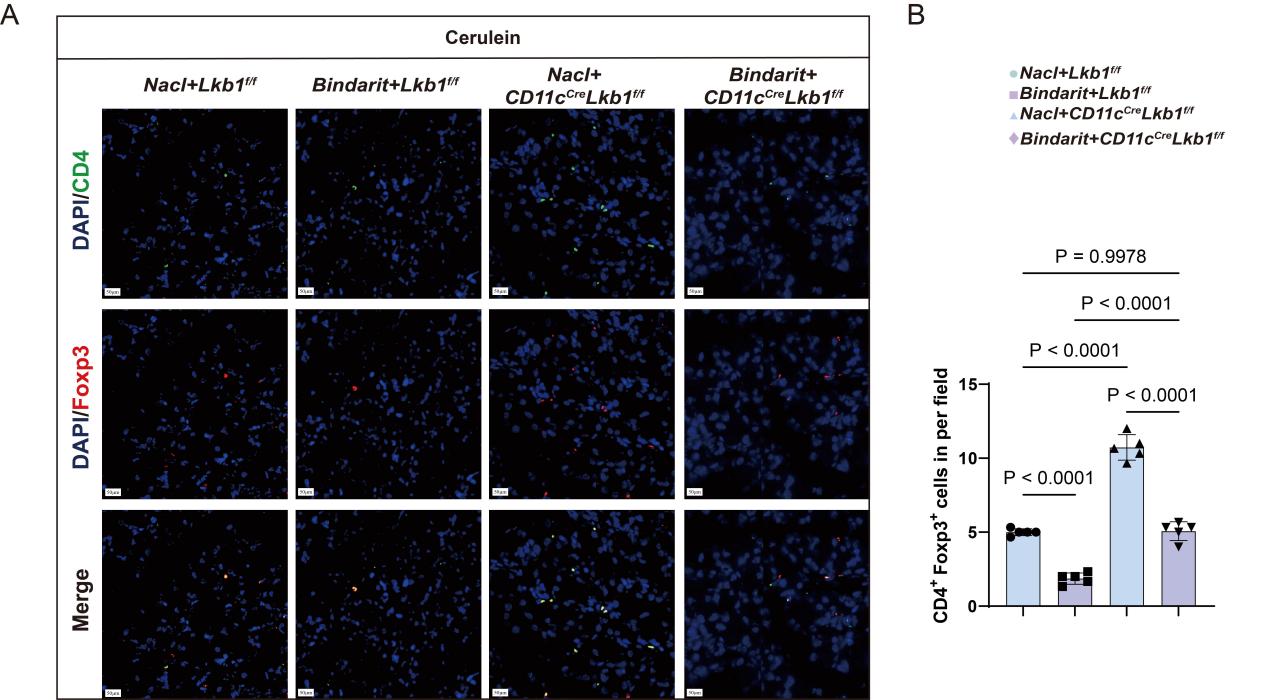


**Figure S9.** Inhibition of CCL2 significantly reduced the accumulation of Treg cells associated with *Lkb1* deletion.

**(A, B)** Immunofluorescence images showing co-staining of CD4, Foxp3, and DAPI in mouse pancreatic tissue. Scale bar, 50 µm. *n* = 5. Data are means ± SEM. Data were analyzed using one-way ANOVA with the Tukey test.


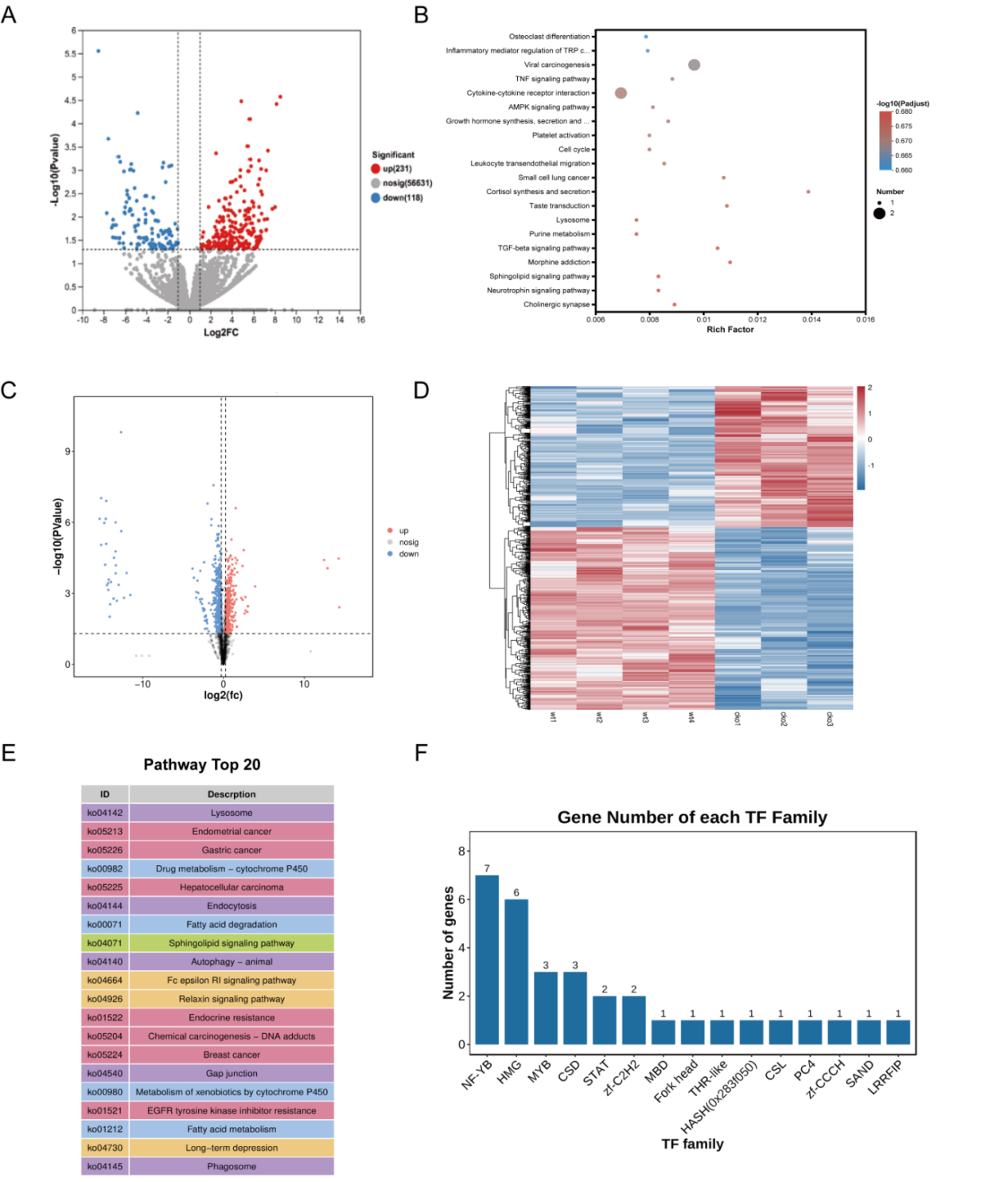


**Figure S10.** Proteomic and transcriptomic sequencing analysis of CD11c^+^ cells.

**(A)** Volcano plot of differentially expressed genes. **(B)** KEGG enrichment analysis of upregulated differentially expressed genes. **(C)** Volcano plot of differentially expressed proteins. **(D)** Heatmap of differentially expressed proteins. **(E)** Enrichment analysis of differentially expressed proteins. **(F)** Prediction of transcription factor (TF) families from proteomic data. (A, B) *n* = 4, (C-F) *Lkb1^f/f^* (*n* = 4) and *CD11c^Cre^Lkb1^f/f^* (*n* = 3) mice.

**
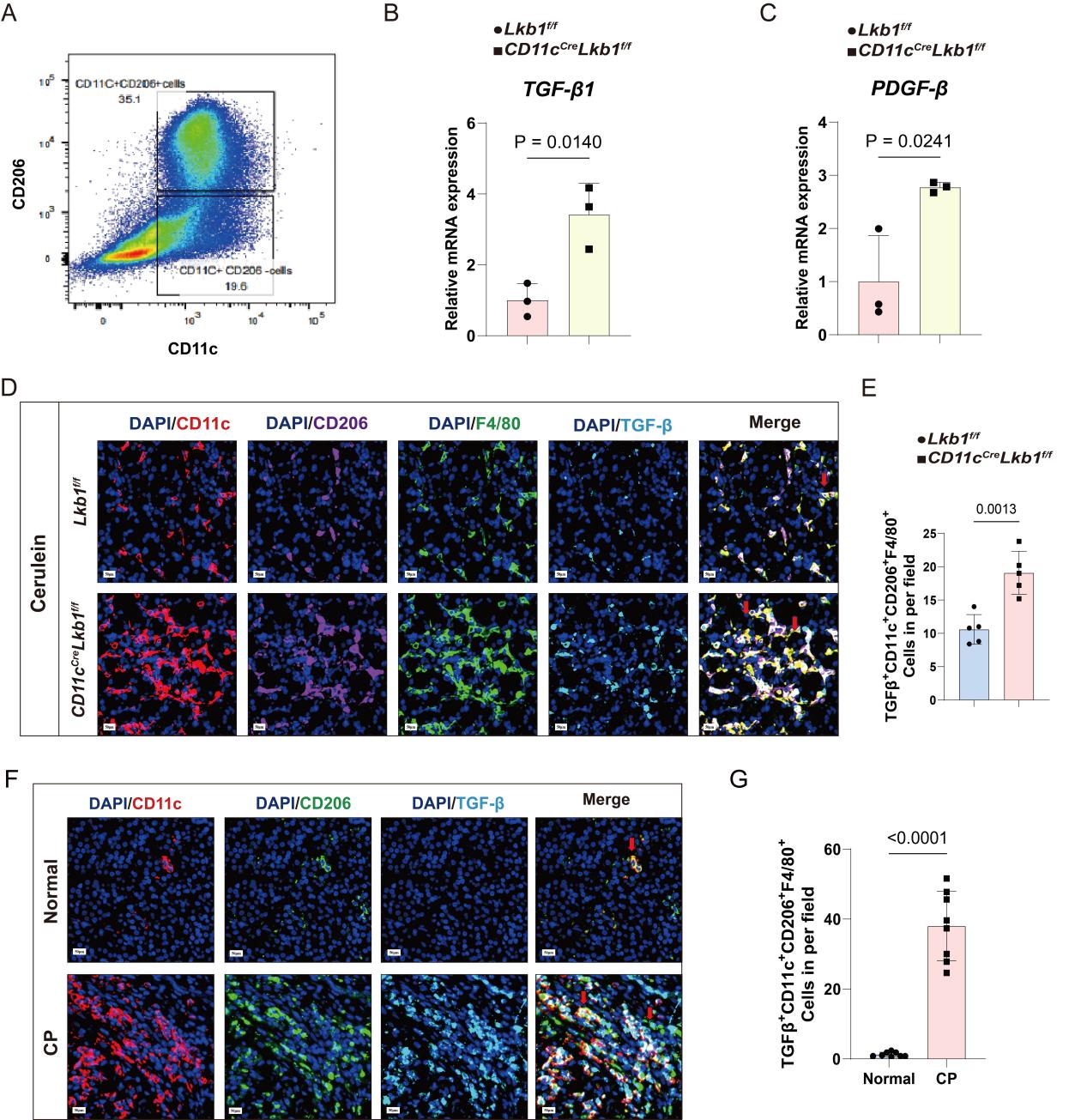
**

**Figure S11.** *Lkb1* deletion in CD11c^+^ cells promotes the expression of CD206 and profibrotic proteins.

**(A)** Representative flow cytometry plots and percentages of CD11c^+^CD206^+^ cells. **(B, C)** mRNA expression levels of *TGF-β1, PDGF-β* in CD11c^+^ cells. *n* = 3. **(D, E)** Representative immunofluorescence images showing co-staining for CD11c, CD206, F4/80, TGF-β, and DAPI in mouse pancreatic tissue. Scale bar, 50 µm. *n* = 5. **(F, G)** Immunofluorescence images showing co-staining of CD11c, CD206, TGF-β and DAPI in human pancreatic tissue. Scale bar, 50 µm. *n* = 8. Data are means ± SEM. Unpaired Student’s t tests were used to evaluate statistical signiﬁcance.


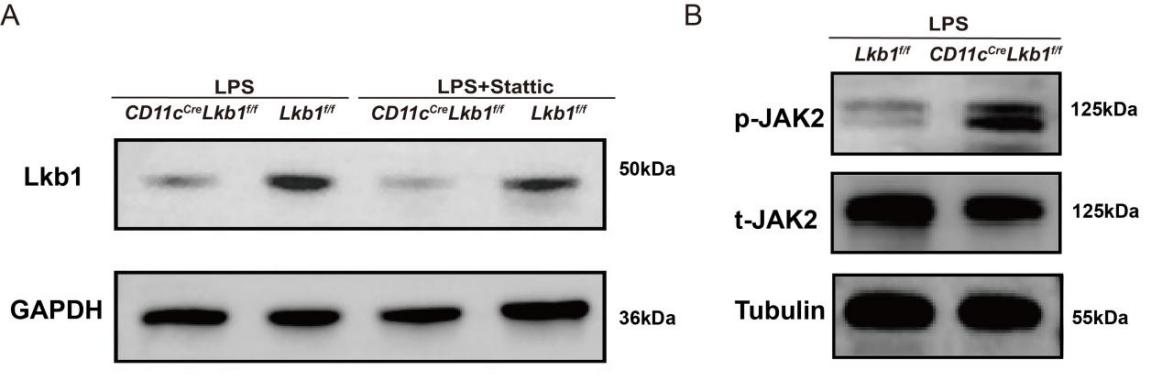


**Figure S12.** *Lkb1* regulates the expression of its target gene *CCL2* in CD11c^+^CD206^+^ macrophages by inhibiting STAT3 phosphorylation.

**(A)** Western blot analysis of Lkb1 in CD11c^+^CD206^+^ macrophages. **(B)** Western blot analysis of p-JAK2 and JAK2 in CD11c^+^CD206^+^ macrophages. (A, B) *n* = 3.


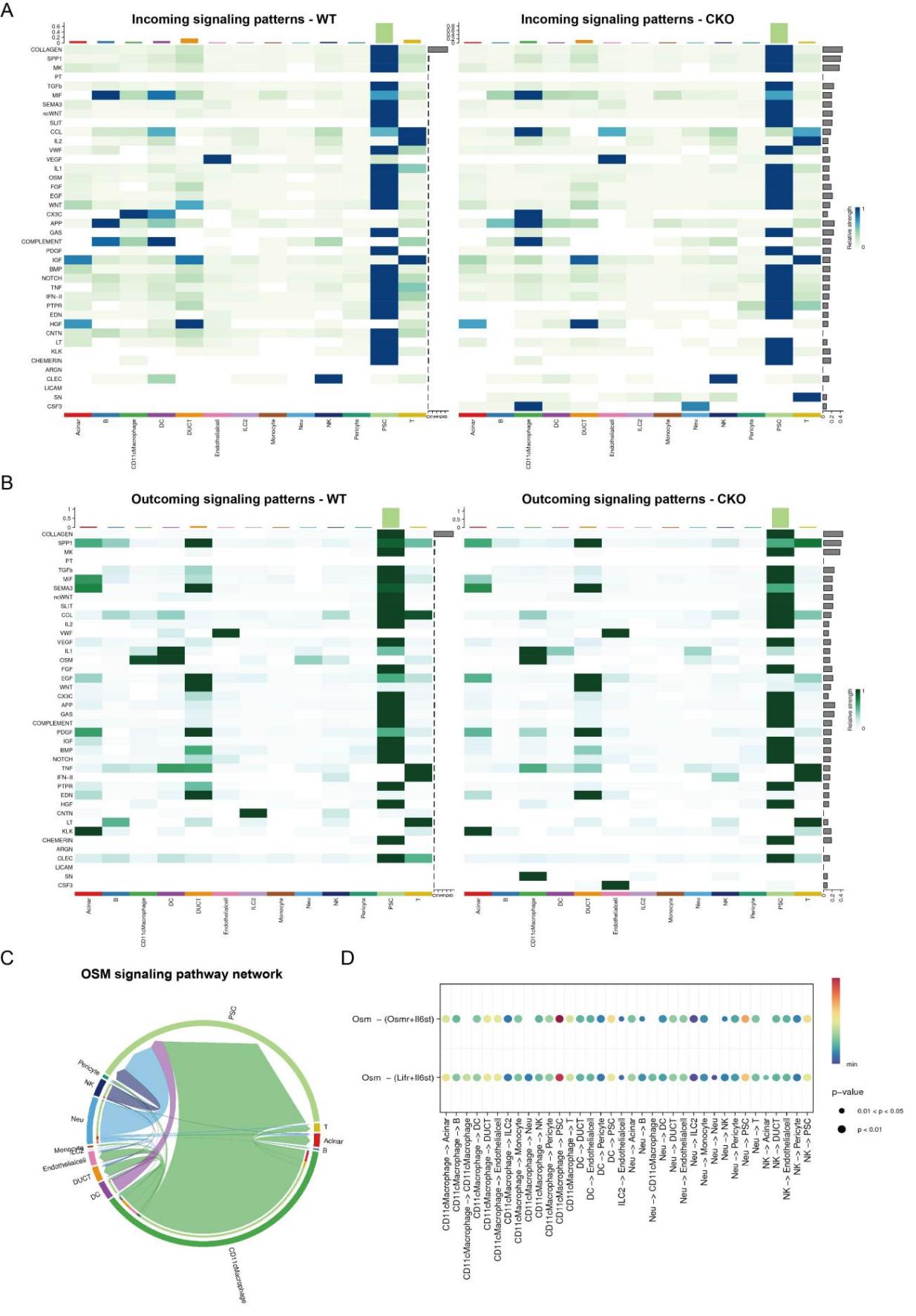


**Figure S13.** The OSM signaling pathway mediates the interaction between CD11c^+^ macrophages and PSCs.

**(A)** Heatmap of incoming signaling pathway levels per cell population between the two groups. **(B)** Heat map of the level of outgoing signaling pathways per cell population between the two groups. **(C)** Circos map of OSM signaling pathway level cellular communication. **(D)** Bubble map of OSM signaling pathway-associated ligand receptor pair horizontal cellular communication. (A-D) *Lkb1^f/f^* (*n* = 4) and *CD11c^Cre^Lkb1^f/f^* (*n* = 3) mice.


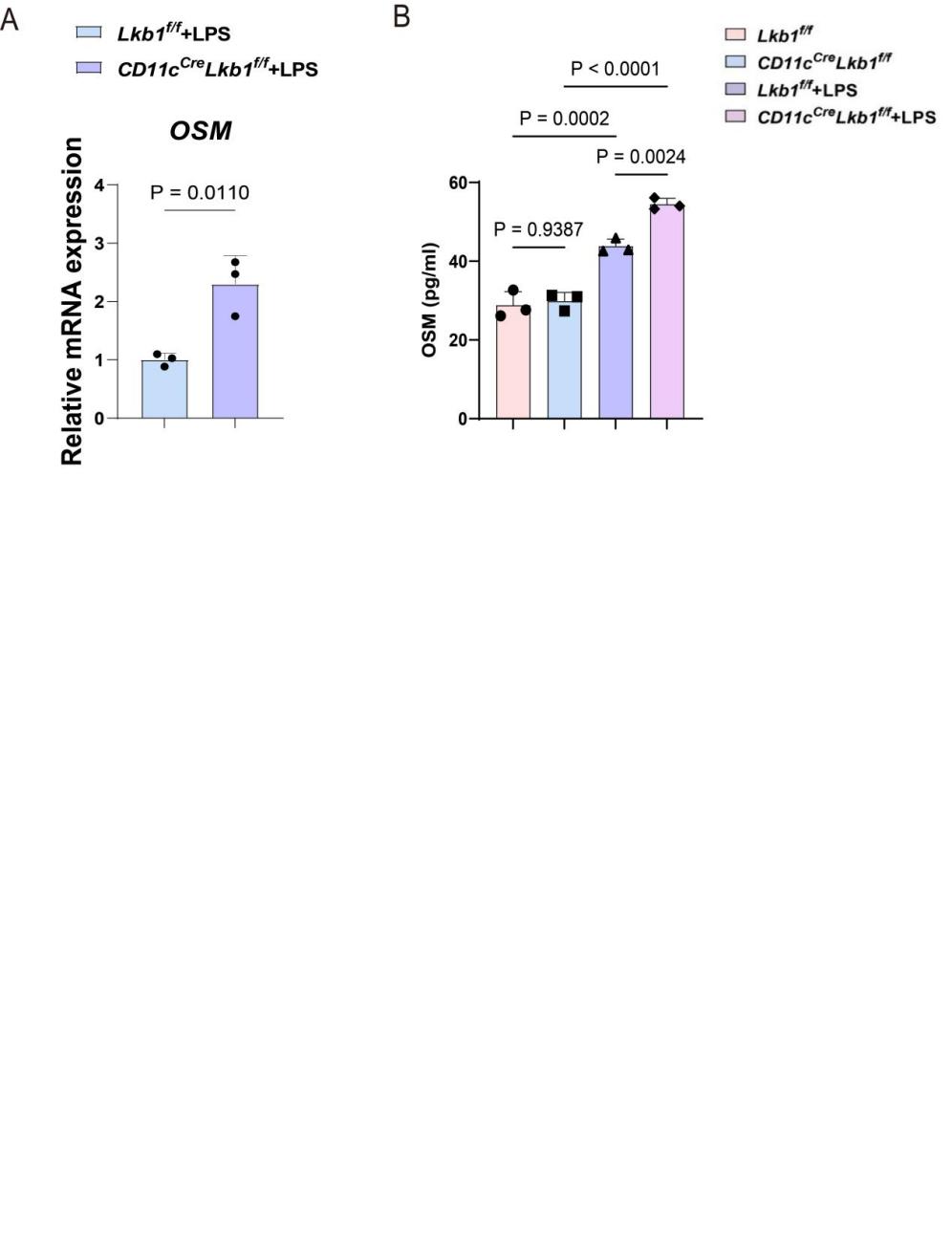


**Figure S14.** *Lkb1* mediates elevated OSM expression through its downstream targets SIK and MARK.

1. mRNA expression levels of *OSM* in CD11c^+^CD206^+^ macrophages. **(B)** OSM secretion level in cell culture supernatants. (A-B) *n* = 3. Data are means ± SEM. Unpaired Student’s t tests (A) were used to evaluate statistical signiﬁcance. Data were analyzed using one-way ANOVA (B) with the Tukey test.


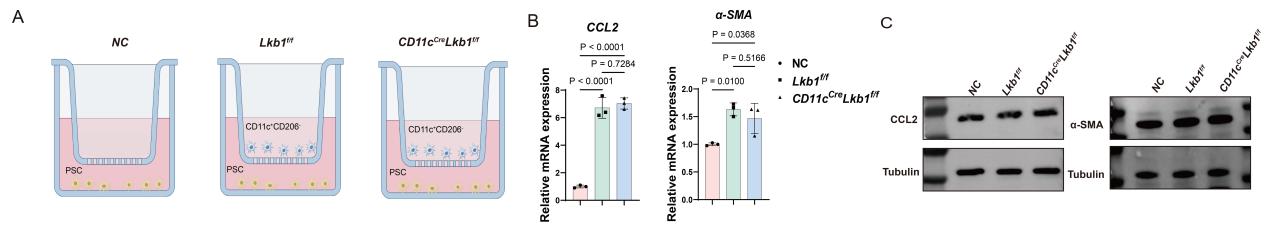


**Supplementary Figure 15.** *Lkb1* deletion in CD11c^+^CD206^-^ cells had no effect on PSC activation or CCL2 secretion.

**(A)** Schematic diagram of the co-culture system. **(B)** mRNA expression levels of *CCL2* and *α-SMA* in PSCs following co-culture. **(C)** Western blot analysis of CCL2 and α-SMA in PSCs following co-culture. (A-C) *n* = 3. Data are presented as means ± SEM. Data were analyzed using one-way ANOVA with the Tukey test.


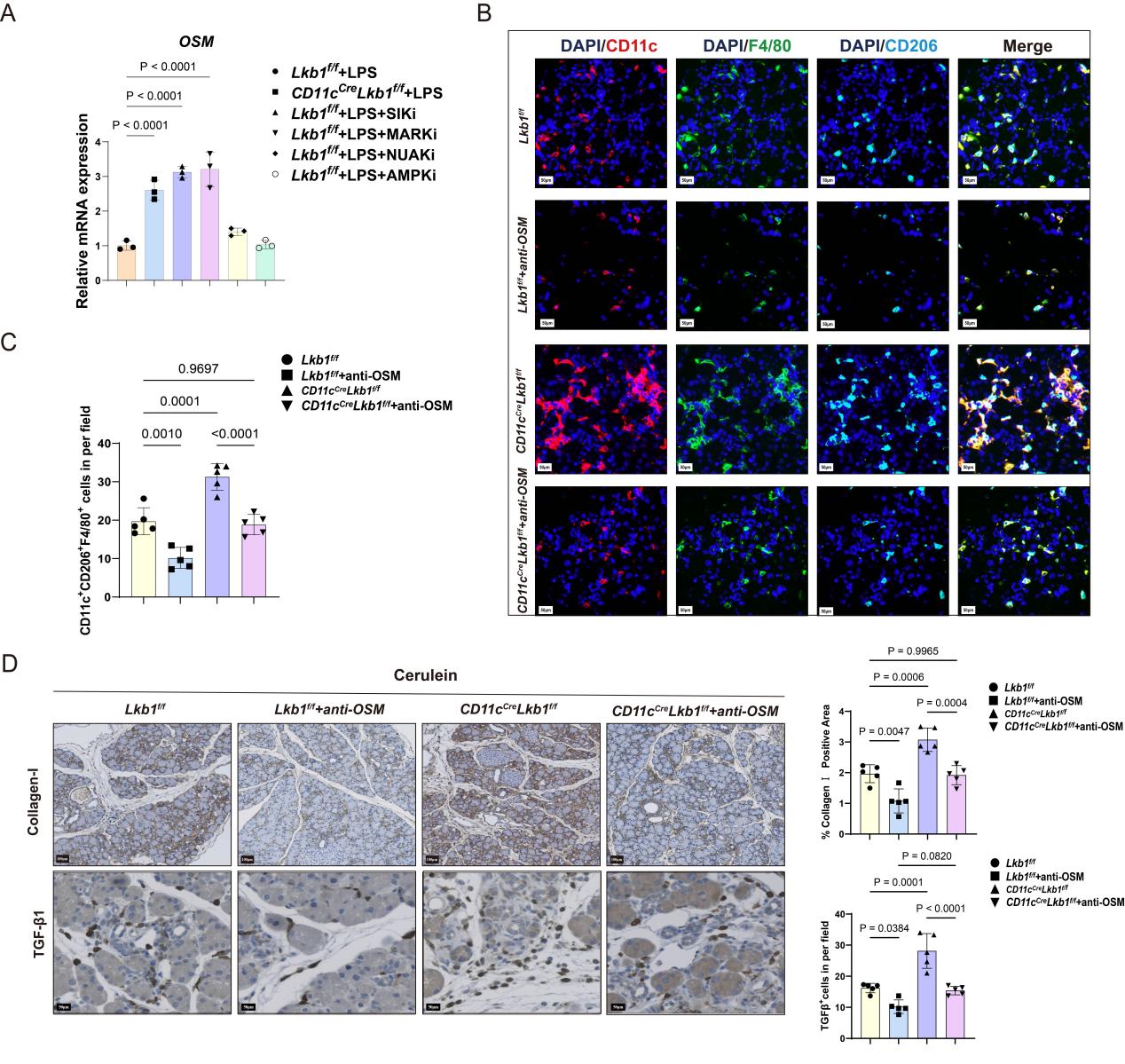


**Figure S16.** Anti-OSM neutralizing antibody reversed the increased CD11c^+^CD206^+^ macrophage infiltration caused by *Lkb1* deletion in CD11c^+^ cells.

**(A)** mRNA expression levels of *OSM* in PSCs pretreated with SIK (500 nmol/mL), MARK (50 μmol/mL), NUAK (10 μmol/mL), AMPK (10 μmol/mL) inhibitors. **(B, C)** Representative immunofluorescence images showing co-staining for CD11c, CD206, F4/80 and DAPI in mouse pancreatic tissue. Scale bar, 50 µm. *n*=5. **(D)** Representative IHC staining images and statistical analysis of collagen-I (scale bar, 100 µm) and TGF-β1 (scale bar, 50 µm)， *n* = 5. Data were analyzed using one-way ANOVA with the Tukey test.
